# An Essential Role of Variant Histone H3.3 for Ectomesenchyme Potential of the Cranial Neural Crest

**DOI:** 10.1371/journal.pgen.1002938

**Published:** 2012-09-20

**Authors:** Samuel G. Cox, Hyunjung Kim, Aaron Timothy Garnett, Daniel Meulemans Medeiros, Woojin An, J. Gage Crump

**Affiliations:** 1 Department of Cell and Neurobiology, Eli and Edythe Broad Center for Regenerative Medicine and Stem Cell Research, University of Southern California Keck School of Medicine, Los Angeles, California, United States of America; 2 Department of Biochemistry, Norris Comprehensive Cancer Center, University of Southern California Keck School of Medicine, Los Angeles, California, United States of America; 3 Department of Ecology and Evolutionary Biology, University of Colorado, Boulder, Colorado, United States of America; University of Washington, United States of America

## Abstract

The neural crest (NC) is a vertebrate-specific cell population that exhibits remarkable multipotency. Although derived from the neural plate border (NPB) ectoderm, cranial NC (CNC) cells contribute not only to the peripheral nervous system but also to the ectomesenchymal precursors of the head skeleton. To date, the developmental basis for such broad potential has remained elusive. Here, we show that the replacement histone H3.3 is essential during early CNC development for these cells to generate ectomesenchyme and head pigment precursors. In a forward genetic screen in zebrafish, we identified a dominant D123N mutation in *h3f3a*, one of five zebrafish variant histone H3.3 genes, that eliminates the CNC–derived head skeleton and a subset of pigment cells yet leaves other CNC derivatives and trunk NC intact. Analyses of nucleosome assembly indicate that mutant D123N H3.3 interferes with H3.3 nucleosomal incorporation by forming aberrant H3 homodimers. Consistent with CNC defects arising from insufficient H3.3 incorporation into chromatin, supplying exogenous wild-type H3.3 rescues head skeletal development in mutants. Surprisingly, embryo-wide expression of dominant mutant H3.3 had little effect on embryonic development outside CNC, indicating an unexpectedly specific sensitivity of CNC to defects in H3.3 incorporation. Whereas previous studies had implicated H3.3 in large-scale histone replacement events that generate totipotency during germ line development, our work has revealed an additional role of H3.3 in the broad potential of the ectoderm-derived CNC, including the ability to make the mesoderm-like ectomesenchymal precursors of the head skeleton.

## Author Summary

The evolution of the vertebrate head was made possible in large part by the emergence of a new cell population, the cranial neural crest. These cells contribute to diverse structures of the head, including most of the skull, yet how neural crest cells acquire such broad potential during development has remained a mystery. By studying mutant zebrafish that lack the neural-crest-derived skull, we find that the unusual potential of these cells depends on an “H3.3” version of one of the histone proteins that package their DNA. We propose then that a dramatic change in the packaging of DNA is a key step in allowing crest cells to make a wide range of new cell types in the vertebrate head.

## Introduction

The development of multipotent, migratory NC cells was a key step in the evolution of many of the vertebrate-specific features of the head [1]. CNC cells generate the skeleton of the face and anterior skull, as well as supporting development of the brain and sense organs. NC arises from NPB ectoderm and gives rise to neurons, glia, and pigment cells along nearly the entire anterior-posterior extent of the embryo, yet CNC has a greater potential than trunk NC to generate ectomesenchymal cell types such as facial skeletal precursors [2]–[5]. Whereas we know more about how neuroglial and pigment cell types are specified, how the CNC is able to generate ectomesenchymal derivatives has remained unclear [6]. In particular, the ectomesenchyme derives from the ectoderm yet shares both gene expression signatures and skeletogenic potential with the embryonic mesoderm, suggesting that there may be a large-scale fate transition during its development.

The post-translational modification of histones is emerging as an important mechanism for regulating cell potential during development. In the embryo, early progenitors exhibit a broad potential with cis-regulatory elements for developmental genes existing in a poised state characterized by bivalent histone modifications [7]–[9] and open permissive chromatin structure [10]. A number of proteins that regulate chromatin structure are known to play essential roles in NC development, including CHD7-PBAF [11], [12] and jmjD2A [13]. The CHD7-PBAF chromatin remodeler complex has been shown to bind H3K4me1-positive enhancers of some early CNC genes, and histone demethylase jmjD2A was found to bind regulatory regions proximal to similar early NC genes and was associated with demethylation of repressive H3K9me3 marks. Disruption of CHD7-PBAF and jmjD2A function similarly resulted in reduced expression of early CNC gene expression and defects in NC derivatives, with no effect on upstream NPB/dorsal-neural-tube factors. However, these types of chromatin remodeling complexes also have more general roles in embryonic development [14]; for example CHD7 depletion also results in neural and placodal defects [11], [15]. In contrast, our genetic studies reveal a more selective role of the replacement histone H3.3 in CNC development.

Histone H3 proteins contribute to the fundamental packing unit of chromatin, the nucleosome, which consists of two molecules each of histones H2A, H2B, H3 and H4, wrapped by two turns of double-stranded DNA. Whereas canonical H3 histones (H3.1 and H3.2) are incorporated into chromatin predominantly during replication, H3.3 is also incorporated outside of replication [16], which has implicated it in various histone replacement events including gene regulation. In mammals, H3.3 has been associated with large-scale histone replacement during the specification of primordial germ cells [17] and the inactivation of meiotic sex chromosomes [18]. However, a developmental requirement for H3.3 outside the germ line has yet to be described. Flies lacking both *H3.3* genes are infertile yet largely adult viable with no specific developmental abnormalities [19], [20]. Similarly, mice hypomorphic for *H3.3A* display growth reduction and infertility but no specific developmental defects [21]. However, the presence of multiple, identical copies of histone genes, such as H3.3, has complicated loss-of-function studies, particularly in vertebrates. Through genetic studies in zebrafish, we have identified a D123N mutant form of H3.3 that allows us to dominantly interfere with H3.3 chromatin incorporation during development. In so doing, we have found that the formation of CNC cells, and their subsequent lineage potential, are particularly sensitive to defects in H3.3 incorporation.

## Results

### A dominant H3.3 mutation specifically disrupts CNC development

**Figure 1:**
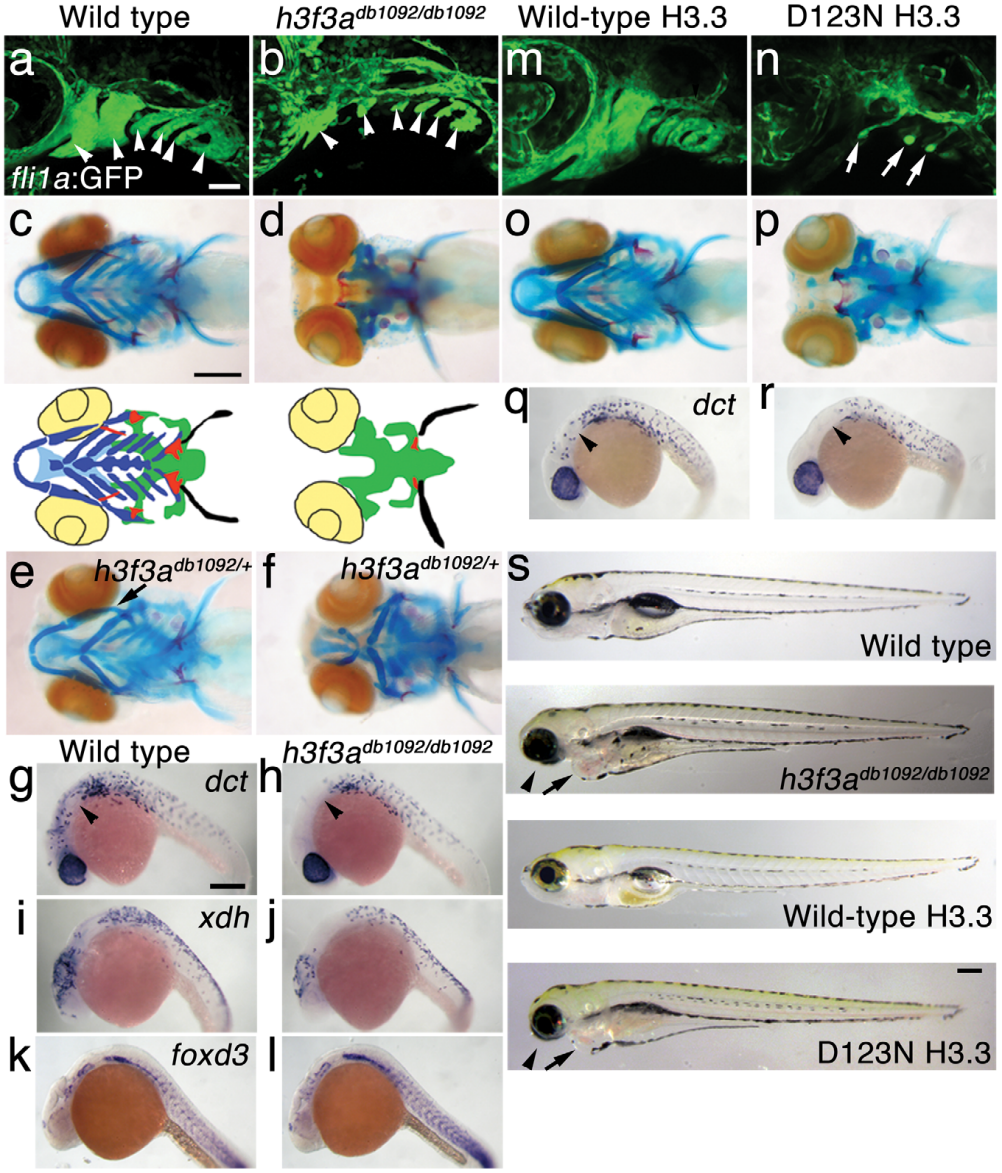
A dominant H3.3 mutation results in losses of CNC–derived head skeleton and pigment cells. a, b, *fli1a*:GFP-labeled arch ectomesenchyme (arrowheads) is greatly reduced, yet *fli1a*:GFP-positive endothelial cells (top) are unaffected, in both homozygous and heterozygous *h3f3a^db1092^* mutants at 34 hpf (7/7 mutant; 0/10 wild-type). c, d, Homozygous *h3f3a^db1092/db1092^* embryos specifically lack the CNC-derived head skeleton at 5 dpf (36/71 complete loss; 35/71 partial loss). Diagrams show the CNC-derived cartilage (blue) and bone and teeth (red), mesoderm-derived cartilage (green), pectoral fin cartilage (black), and eyes (yellow). e, f, *h3f3a^db1092/+^* heterozygous larvae exhibit a wide range of craniofacial defects. In some cases, no defects are observed in the facial skeleton and heterozygotes are adult viable (not shown). In mild cases (e), dorsal cartilage and bone of the first and second arches are preferentially reduced, including the dorsal hyosymplectic cartilage and opercular bone of the second arch (arrow). In more severe cases (f), the cartilage and bone of the first arch and dorsal second arch are greatly reduced, with the anterior neurocranium and the posterior ceratobranchial cartilages being less affected. The frequency of skeletal phenotypes in *h3f3a^db1092/+^* heterozygous larvae is highly variable between clutches. g, h, At 27 hpf, *dct*-positive melanophore precursors are selectively missing anterior to the ear (arrowheads) in both homozygous and heterozygous *h3f3a^db1092^* embryos (5/5 mutant; 0/5 wild-type). i, j, At 27 hpf, cranial *xdh*-positive xanthophore precursors are mildly reduced in both homozygous and heterozygous *h3f3a^db1092^* embryos (6/8 mutant; 0/4 wild-type). k, l, Wild-type and both homozygous and heterozygous *h3f3a^db1092^* embryos have comparable numbers of *foxd3*-positive glial cells at 24 hpf (4 mutant; 5 wild-type). m-r, D123N *h3f3a* mRNA-injected but not wild-type *h3f3a* mRNA-injected embryos lack *fli1a*:GFP-positive ectomesenchyme (9/19 D123N; 0/12 wild-type), CNC-derived head skeleton (24/45 D123N; 0/21 wild-type) and cranial *dct*-positive melanophore precursors (anterior to the ear: arrowheads) (7/14 D123N; 0/11 wild-type). *fli1a*:GFP-positive blood vessels are unaffected (arrows). s, Except for the loss of the majority of the skull (no facial structures below the level of the eye: arrowheads) and mild heart edema (arrows), the overall morphologies of wild-type, homozygous and heterozygous *h3f3a^db1092^*, wild-type *h3f3a*-injected, and D123N *h3f3a*-injected larvae are indistinguishable at 5 dpf. Melanophores (black) and xanthophores (yellow) are also largely normal. Except for panels e and f, homozygous *h3f3a^db1092^* examples are shown. Scale bars: a, b, m & n, 50 µm; c–l, o–s, 250 µm.

In an ethylnitrosourea mutagenesis screen, we identified a dominant zebrafish mutant, *db1092*, that exhibited severe reductions in *fli1a*:GFP-labeled ectomesenchyme [22] at 36 hours-post-fertilization (hpf) (Figure 1a, 1b). As in other vertebrates, the facial skeleton and anterior skull of the larval zebrafish derive from CNC ectomesenchyme, with the posterior skull being of mesoderm origin [23], [24]. In *db1092* homozygous mutants, nearly all of the CNC-derived cartilage, bone, and teeth were lost at 5 days-post-fertilization (dpf), leaving only the mesoderm-derived skull (Figure 1c, 1d). These skeletal phenotypes were very reminiscent of those seen in *foxd3*; *tfap2a* compound mutants that completely lack CNC, again confirming the CNC specificity of the head skeletal defects in *db1092* mutants [25]. *db1092* homozygous larvae die by around 7 dpf, presumably due to an inability to feed. Whereas some *db1092* heterozygotes survived to adulthood, others exhibited variable reductions of the jaw-support skeleton (Figure 1e, 1f). Due to the shared phenotypes of *db1092* homozygous and heterozygous embryos, “*db1092* mutants” will refer to both genotypes unless otherwise stated.

We next examined whether other NC derivatives, such as pigment cells, glia, and neurons, were affected in *db1092* mutants. Melanophore pigment cells and their *dct*-positive precursors were reduced in the cranial but not trunk regions of *db1092* mutants, and to a lesser extent so were xanthophore pigment cells and their *xdh*-positive precursors (Figure 1h, 1j and Figure S1). In contrast, *foxd3*-positive peripheral glia (Figure 1l), neurons of the cranial ganglia (Figure S1), and the dorsal root ganglia and sympathetic neurons derived from trunk NC (data not shown) were unaffected. *db1092* mutants also displayed mild heart edema, consistent with a known CNC contribution to the heart [26], but had an otherwise remarkably normal morphology at 5 dpf (Figure 1s). In summary, *db1092* mutants have highly specific reductions of CNC derivatives, in particular the ectomesenchymal/skeletal components of the head.

**Figure 2:**
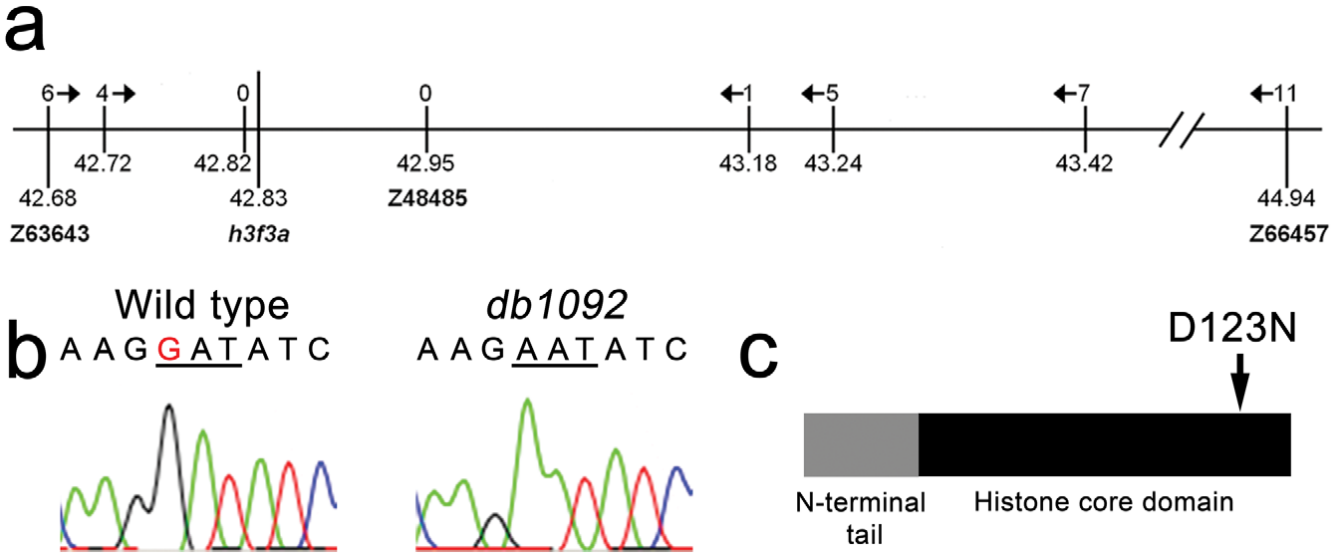
Identification of the *h3f3a^db1092^* lesion. a, The *db1092* allele was crossed to the highly polymorphic WIK strain for linkage analysis. As the *db1092* mutation is semi-dominant, we enriched for putative heterozygotes by selecting for partial head skeletal loss, and events were scored as recombination only if both chromosomes displayed the wild-type WIK polymorphism. Using a set of microsatellite ‘Z’ markers spanning the zebrafish genome, we placed *db1092* on linkage group 3 near Z3725 and Z20058, and subsequent linkage analysis placed it between Z63643 and Z66457. Recombinants per 1065 meioses are listed above each marker. Sequencing of 3′ UTRs identified single nucleotide polymorphisms (SNPs) that created or destroyed restriction sites between the mutant and WIK chromosomes. These SNPs (identified by their position in millions of base pairs) and Z48485 were then used to map *db1092* to a 464 kb interval. b, Electrophoretograms show a G to A transition in the *h3f3a* gene of *db1092* homozygotes. c, Schematic of the H3.3 variant histone protein encoded by *h3f3a*. The *db1092* mutation results in a D123N substitution near the C-terminus of the core domain.

**Figure 3:**
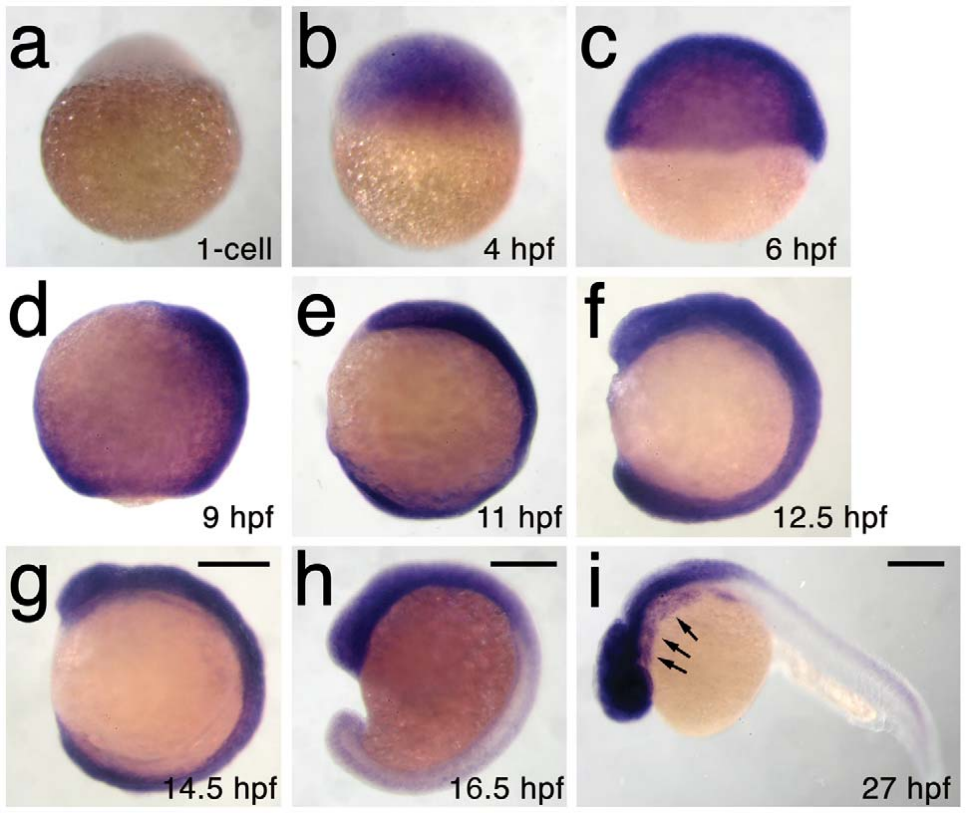
*h3f3a* is ubiquitously expressed throughout embryogenesis. a–i, Lack of *h3f3a* expression at the one-cell stage shows that *h3f3a* mRNA is not maternally provided. From 4–14.5 hpf, *h3f3a* is expressed ubiquitously throughout the embryo. By 16.5 hpf and 27 hpf, *h3f3a* expression is still widespread, with higher levels apparent in the anterior part of the embryo, including the CNC-derived ectomesenchyme of the pharyngeal arches (arrows) at 27 hpf. Scale bars = 250 µm.

We next used microsatellite polymorphism mapping to place *db1092* within a 464 kb region on linkage group 3 which contained *h3f3a*, one of five genes encoding identical H3.3 proteins (Figure 2). Sequencing of *h3f3a* revealed a G to A transition in *db1092* that converts aspartic acid 124 to asparagine (referred to as D123N due to cleavage of the amino-terminal methionine). Given the semi-dominant nature of *db1092*, we reasoned that the D123N mutation might result in a dominantly acting version of H3.3. To test this, we separately injected mRNAs encoding wild-type and D123N forms of H3.3 into one-cell-stage zebrafish embryos. Whereas wild-type H3.3 had no effect on CNC development, D123N H3.3 caused nearly identical losses of *fli1a*:GFP-positive ectomesenchyme (Figure 1n), CNC-derived head skeleton (Figure 1p), and cranial melanophore precursors (Figure 1r) as seen in the *db1092* mutant, confirming D123N H3f3a as the causative mutation. As reported for other H3.3 genes in zebrafish [27], we found that *h3f3a* was ubiquitously expressed starting at 4 hpf and continuing through 14.5 hpf when CNC has been specified (Figure 3). At 16.5 and 27 hpf, *h3f3a* expression remained largely ubiquitous but was more prominent in the anterior embryo. As both the endogenous *h3f3a^db1092^* gene product, and in particular the mRNA-injected D123N H3.3, are present uniformly throughout the embryo at CNC specification stages, the remarkable specificity of the ectomesenchyme defect is not due to a preferential expression of this particular *h3f3a* gene in CNC precursors. Instead, our data indicate that CNC and ectomesenchyme development are uniquely sensitive to altered H3.3 function.

### Mutant D123N H3.3 dominantly interferes with H3.3 function through aberrant homodimer formation

**Figure 4:**
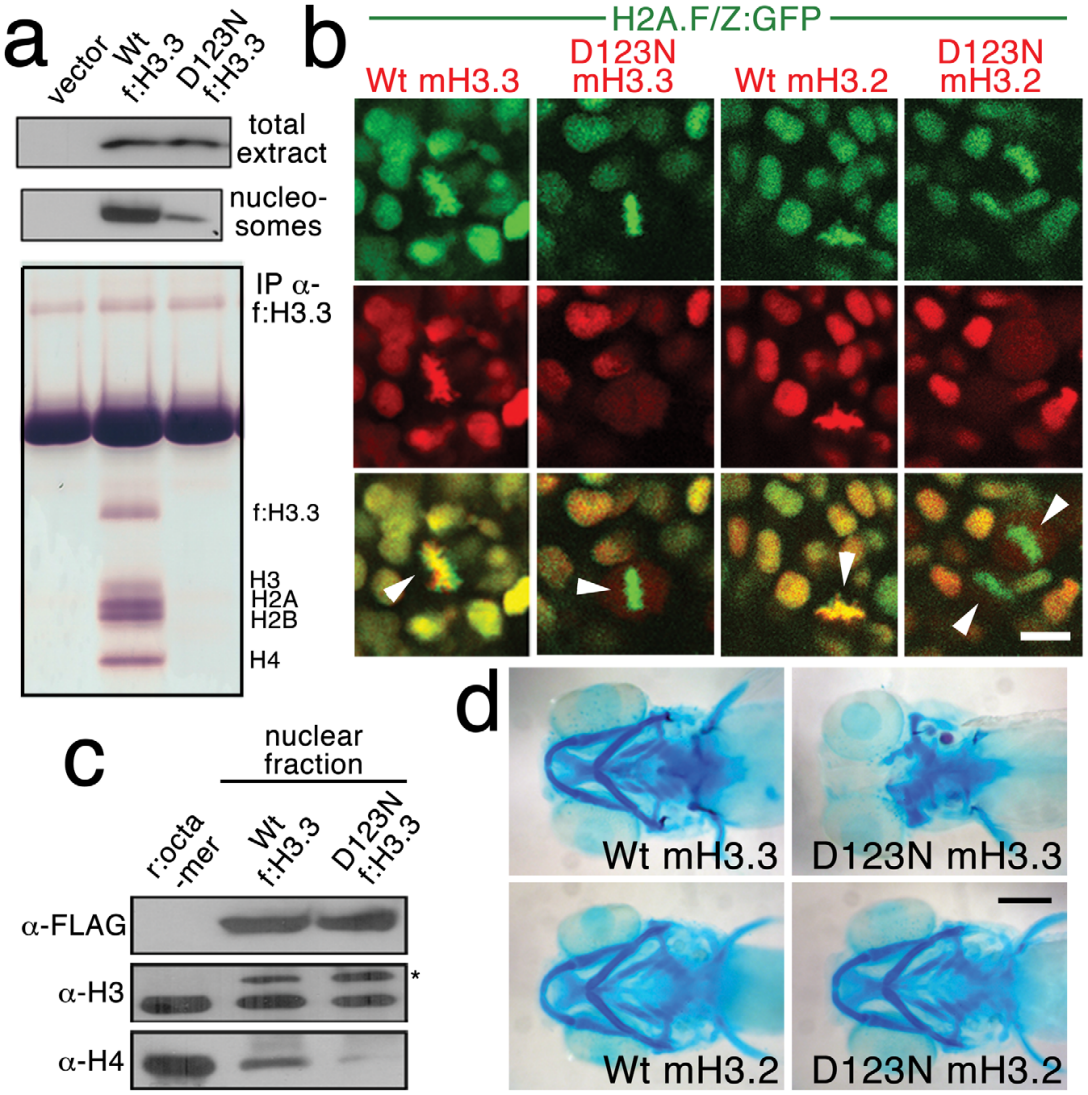
The dominant D123N mutation prevents chromatin incorporation and promotes the formation of aberrant H3 homodimers. a, Western blots show α-FLAG immunostaining of nuclear extracts or purified mononucleosome fractions from HEK cells transfected with vector alone or FLAG-tagged H3.3 (f:H3.3) vectors. Wild-type and D123N f:H3.3 proteins are expressed at equal levels in total extract, but wild-type f:H3.3 is present at much higher levels in the nucleosome fraction (consistent over three replicate experiments). α-FLAG immunoprecipitation from purified nucleosomes shows that wild-type but not D123N f:H3.3 is incorporated into nucleosomes containing H2A, H2B, H3, and H4 (consistent over three replicate experiments). b, Confocal images from H2A.F/Z:GFP embryos expressing wild-type and D123N versions of mCherry(m)H3.3 and mCherry(m)H3.2 fusion proteins. Merged images show that whereas all H3 proteins are nuclear localized in surrounding non-mitotic cells, wild-type mH3.3 and mH3.2, but not D123N mH3.3 and mH3.2, co-localize with H2A.F/Z:GFP in the chromosomes of metaphase/anaphase cells (arrowheads) after nuclear envelope breakdown (wild-type mH3.3, 11/11 cells in 2 embryos; D123N mH3.3, 0/25 cells in 3 embryos; wild-type mH3.2, 21/21 cells in 3 embryos; D123N mH3.2, 0/16 cells in 2 embryos). c, α-FLAG, α-H3 and α-H4 western blots for samples immunoprecipitated by α-FLAG from nuclear extracts of f:H3.3-transfected HEK cells. Recombinant octamer is used as a reference. Whereas both endogenous H3 and H4 co-immunoprecipitate with the wild-type f:H3.3 protein (*), H3 but not H4 co-immunoprecipitates with D123N f:H3.3 (asterisk marks the larger recombinant f:H3.3 protein). Results were consistent over three replicate experiments. d, mRNA injection of D123N mH3.3 (8/17), but not wild-type mH3.3 (0/26), wild-type mH3.2 (0/19), or D123N mH3.2 (0/18), results in loss of the CNC-derived head skeleton at 4 dpf. Scale bars: b, 10 µm; d, 250 µm.

We next investigated the effect of the D123N substitution on H3.3 function. When human embryonic kidney cells were transfected with FLAG-tagged wild-type or D123N H3.3, we found D123N H3.3 to be under-enriched in purified nucleosomes compared to wild-type H3.3 (Figure 4a). The D123N mutation also prevented the incorporation of H3.3 into chromatin in zebrafish embryos. Whereas mCherry-tagged forms of both wild-type and D123N H3.3 were nuclear localized during interphase, during metaphase/anaphase, when the nuclear membrane breaks down and condensed chromosomes are easily distinguished, wild-type but not D123N H3.3 co-localized with chromatin marked by a GFP-tagged H2A.F/Z histone [28]. The failure of D123N H3.3 to associate with chromatin was observed both in the eye (Figure 4b) and in the *pax3a*:GFP-positive NPB precursors of CNC (Figure S2). Time-lapse recordings showed that mCherry-D123N-H3.3 nuclear fluorescence immediately returned upon resumption of interphase, indicating that the diffuse metaphase fluorescence of D123N H3.3 was due to a lack of chromatin incorporation and not degradation (Figure S3).

**Figure 5:**
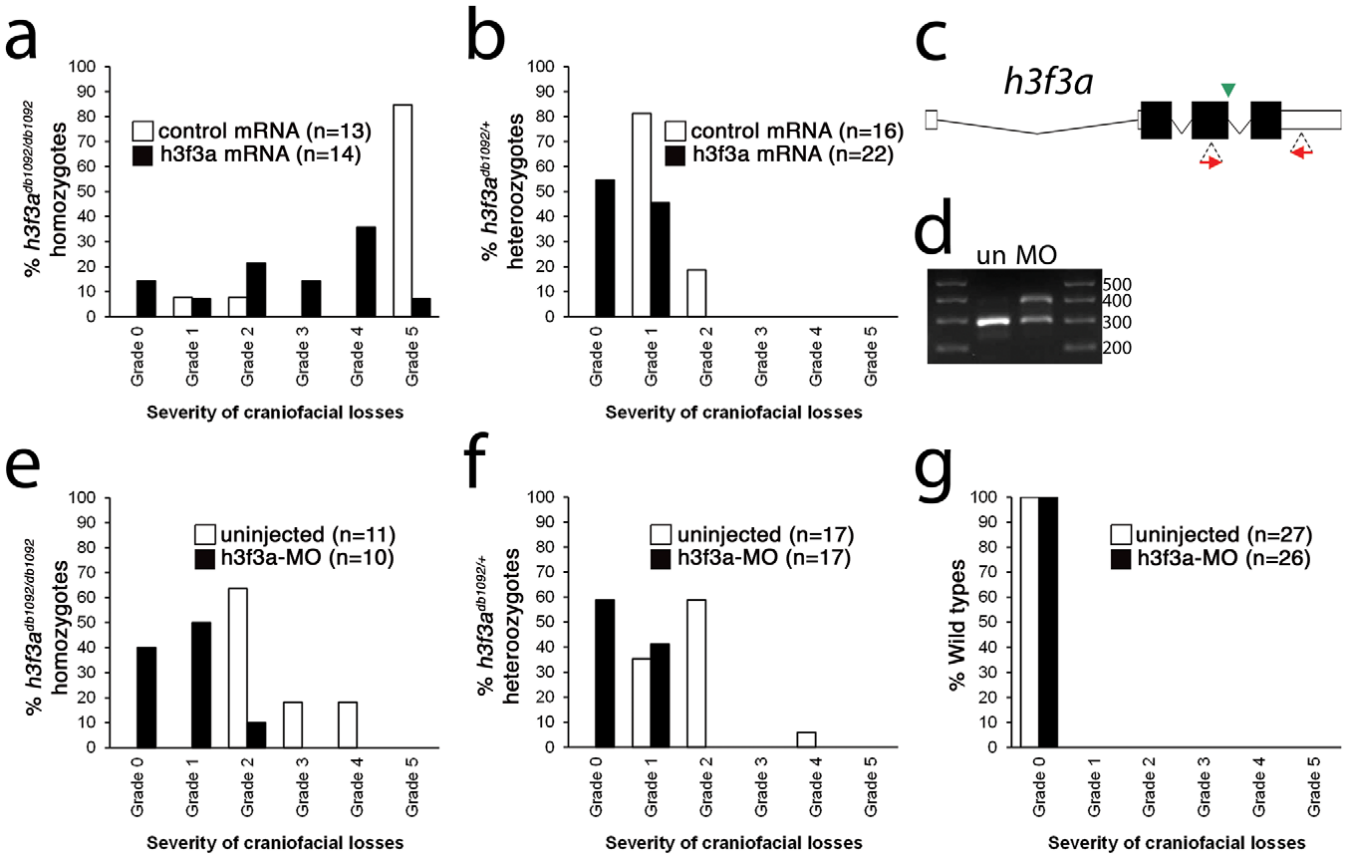
Injection of wild-type H3.3 RNA and reduction of mutant H3.3 levels both rescue craniofacial skeletal development in *h3f3a^db1092^* mutants. a, b, Results from scoring the severity of craniofacial losses in 5 dpf larval head skeletons from *h3f3a^db1092^* siblings injected with 450 ng/µl mRNA encoding wild-type H3.3 or a control kikGR fluorescent protein. The scoring system ranges from Grade 0 (wild-type phenotype) to Grade 5 (complete loss of CNC derivatives); see Materials and Methods for more detail. H3.3-mRNA-injected *h3f3a^db1092^* homozygotes (a) and heterozygotes (b) exhibited a decrease in the severity of *h3f3a^db1092^* craniofacial phenotypes over kikGR-RNA-injected controls (significant by Fisher's exact test: homozygotes, p = 7.7E-05; heterozygotes, p = 1.0E-04). c, An antisense morpholino oligonucleotide was designed to inhibit splicing at the exon 3/intron 3–4 boundary (green arrowhead) of the *h3f3a* transcript. Morpholinos were injected into one-cell-stage *h3f3a^db1092^* embryos at 400 µM. d, Morpholino efficacy was demonstrated by PCR amplification between exons flanking the targeted splice junction from 10 hpf cDNA from 20 pooled embryos (position of primers shown as red arrows in c). Compared to the sample from uninjected (un) embryos, the morpholino-treated sample (MO) exhibited a partial decrease in PCR product representing spliced transcript (295 bp) and a concomitant increase in un-spliced PCR product (390 bp). e, f, Compared to uninjected siblings, both morpholino-injected *h3f3a^db1092^* homozygotes (e) and heterozygotes (f) exhibited a decrease in the severity of *h3f3a^db1092^* craniofacial phenotypes (significant by Fisher's exact test: homozygotes, p = 2.0E-04; heterozygotes, p = 6.3E-06). (g) Craniofacial development in wild-type embryos was unaffected by morpholino injection.

Nucleosome assembly normally proceeds through an H3–H4 heterodimer, an H3–H4/H3–H4 heterotetramer, and then the octamer. The C-terminal domain of H3 forms a 4-helix bundle with a second H3, thus promoting the association of the two H3–H4 pairs within the nucleosome. D123 lies within this domain and forms an intermolecular bond with histidine 113 (H113) of the alternate H3 [29]. In purified nuclear fractions from cultured cells, we found that wild-type H3.3 associated with both H3 and H4, reflecting the presence of heterotetramers and octamers. Remarkably, rather than blocking H3.3-H3 interactions, the D123N mutation resulted in H3.3 forming aberrant associations with H3 in the absence of H4 (Figure 4c). The dominant-negative function of D123N H3.3 would thus be explained by its ability to complex with wild-type H3.3 in the absence of H4, thus interfering with the ability of wild-type H3.3 to be incorporated into nucleosomes. Consistent with this, misexpression of D123A and H113A mutant versions of H3.3, which are predicted to fail to associate with H3, had no effect on CNC development (data not shown). Of note, we were unable to detect mislocalization of mCherry-tagged wild-type H3.3 within *h3f3a^db1092^* homozygotes (Figure S4), suggesting that H3.3 is not whole-scale depleted from chromatin in mutants. Thus, the dominant effects of D123N H3.3 on CNC development could be due either to a partial depletion of wild-type H3.3 from chromatin, which falls below our level of detection, or alternatively a failure to incorporate H3.3 at a particular subset of loci, such as at poised and active enhancers. Importantly though, increasing H3.3 levels by injection of wild-type H3.3 mRNA rescued the head skeletal defects of *h3f3a^db1092^* mutants, showing that defects are indeed due to compromised H3.3 incorporation and not neomorphic effects of mutant D123N H3.3 on unrelated pathways (Figure 5a, 5b).

Whereas misexpression of mutant D123N H3.3 results in severe CNC defects, it remains unclear whether loss of H3.3 genes can cause a similar effect. H3.3 genes are some of the most highly expressed in cells, and we were unable to generate function-blocking morpholinos that substantially reduced H3.3 levels for any of the endogenous genes (data not shown). However, injection of a morpholino that partially reduced splicing of the *h3f3a* gene was able to rescue *h3f3a^db1092^* mutants by reducing D123N H3.3 levels, though it caused no CNC defects on its own (Figure 5c–5g). Mice with hypomorphic mutations in *H3.3A*, one of two H3.3 genes, also do not exhibit specific CNC/craniofacial defects [21]. In one model, the dominant D123N H3.3 protein is particularly effective at depleting available H3.3 levels below the threshold required for CNC development, with a general reduction in H3.3 nucleosome incorporation resulting in CNC defects. Alternatively, as discussed above, CNC defects may arise from defects in a particular subset of nucleosomal H3.3 incorporation. Indeed, recent studies suggest that distinct chaperone complexes load H3.3 at different types of genomic sites, and particular complexes could be differentially sensitive to dominant effects of mutant D123N H3.3 [30]. In the future, the generation of null mutants for some or all H3.3 genes should help address whether CNC development is particularly sensitive to reductions in general or specific modes of H3.3 nucleosomal incorporation, as well as revealing to what extent H3.3 is required in other non-CNC tissues.

### CNC development depends on replication-independent H3.3 incorporation

Histone H3.3 can be incorporated into chromatin both during and outside of DNA replication. In contrast, canonical H3.2 differs from H3.3 at only four amino acids yet incorporates very poorly into chromatin outside of replication [16]. Hence, in order to test the role of replication-coupled loading of H3 histones in CNC development, we performed embryo-wide misexpression of a version of H3.2 carrying the same D123N mutation. As with H3.3, the D123N mutation blocked the incorporation of mCherry-tagged H3.2 into H2A.F/Z:GFP-labeled chromatin (Figure 4b). However, unlike D123N H3.3, D123N H3.2 had no effect on development of *fli1a*:GFP-positive ectomesenchyme or CNC-derived head skeleton (Figure 4d and data not shown). The inability of D123N-H3.2 to inhibit ectomesenchyme formation strongly suggests that CNC development relies on the unique replication-independent mode of H3.3 deposition.

### H3.3 function is required cell-autonomously for CNC but not NPB gene expression

**Figure 6:**
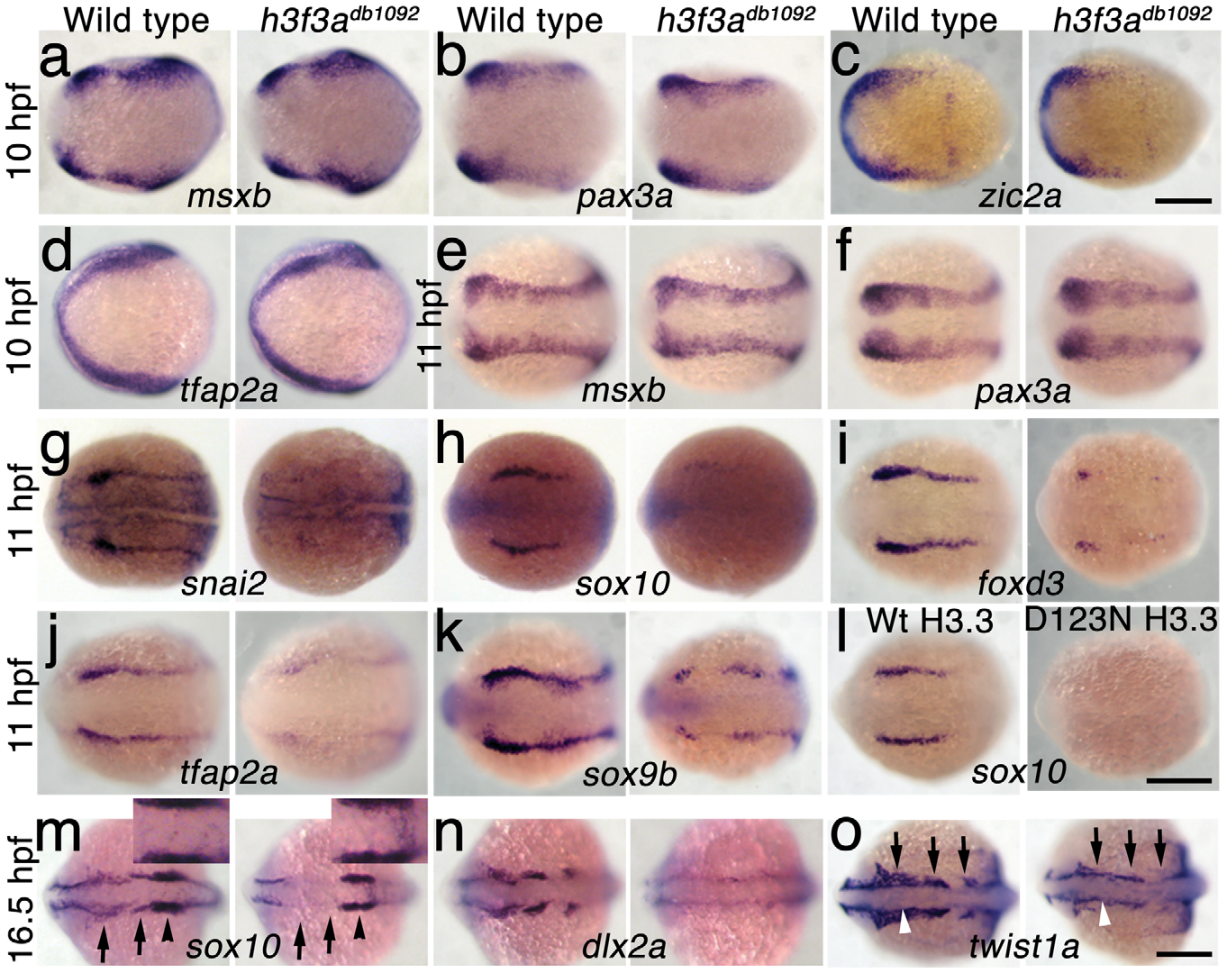
H3.3 functions at the NPB–CNC transition. a–f, Expression of *msxb*, *pax3a*, *zic2a*, and *tfap2a* at 10 hpf and *msxb* and *pax3a* at 11 hpf is indistinguishable between wild types and both homozygous and heterozygous *h3f3a^db1092^* mutants (mut) (n≥10 for each). g–k, At 11 hpf, both homozygous and heterozygous *h3f3a^db1092^* mutants have severe reductions in the expression of *snai2* (4/4 mut; 0/4 wt), *sox10* (10/12 mut; 0/6 wild-type), *foxd3* (9/9 mut; 0/5 wt), *tfap2a* (7/7 mut; 0/4 wt), and *sox9b* (8/8 mut; 0/3 wt). l, *sox10* expression is also lost in embryos injected with D123N (10/12) but not wild-type (0/16) *h3f3a* mRNA. m, In both homozygous and heterozygous *h3f3a^db1092^* embryos, *sox10* expression partially recovers by 16.5 hpf yet is specifically reduced in presumptive CNC ectomesenchyme domains (arrows) (6/6 mut; 0/4 wt). An increase in *sox10*-positive cells is evident in the mutant dorsal neural tube (insert) between the *sox10*-positive otic placodes (arrowheads) which are unaffected in mutants. n, At 16.5 hpf, *dlx2a* expression in three streams of migrating ectomesenchyme is reduced in both homozygous and heterozygous *h3f3a^db1092^* mutants (6/7 mut; 0/5 wt). o, The 16.5 hpf ectomesenchyme expression (arrows) of *twist1a* is reduced in both homozygous and heterozygous *h3f3a^db1092^* mutants yet paraxial mesoderm expression is unaffected (white arrowheads) (8/8 mut; 0/5 wt). In all panels, homozygous *h3f3a^db1092^* examples are shown. All images are dorsal views with anterior to the left. Scale bars: 250 µm.

**Figure 7:**
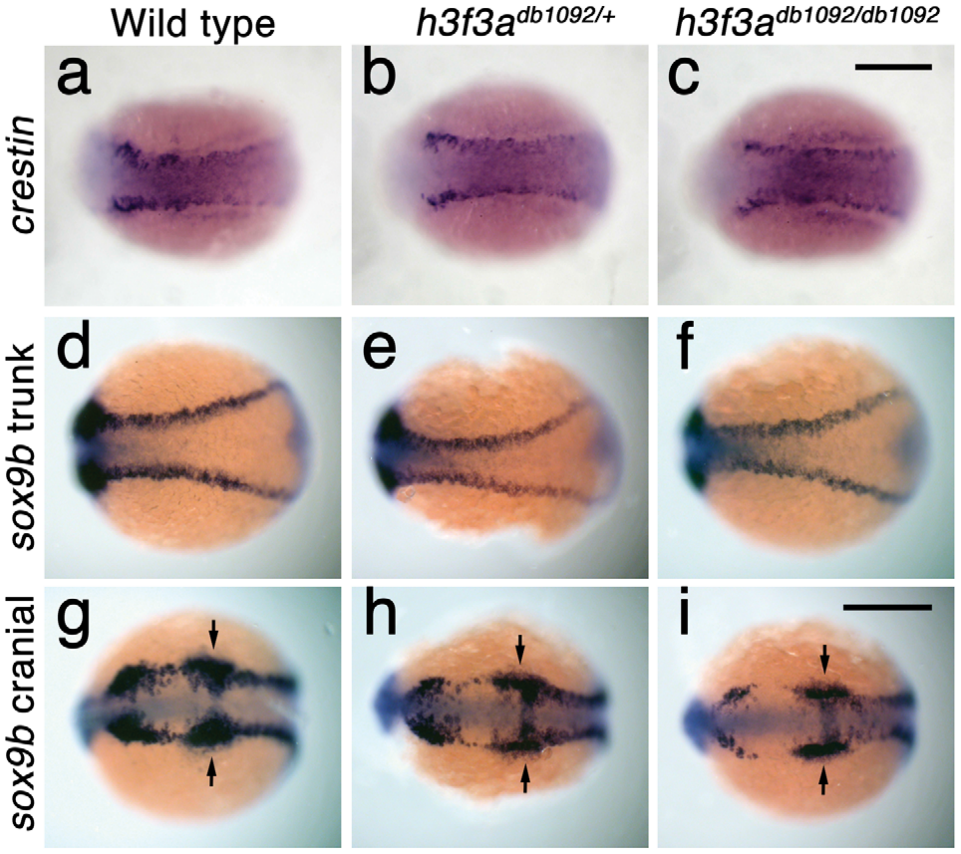
Trunk NC is largely unaffected in *h3f3a^db1092^* mutants. a–c, *crestin* expression at 11.7 hpf shows similar amounts of trunk NC in wild-type and both homozygous and heterozygous *h3f3a^db1092^* embryos (n = 5 for each genotype). d–i, Trunk views of *sox9b* expression at 11.7 hpf show that trunk NC specification is largely normal in both homozygous and heterozygous *h3f3a^db1092^* mutants (n = 10 for each genotype). Cranial views of the same embryos show reduced amounts of *sox9b*-expressing CNC. Arrows show the *sox9b*-positive otic placodes that are unaffected in mutants. Scale bars = 250 µm.

In order to understand at what level H3.3 functions in CNC development, we next examined gene expression in *h3f3a^db1092^* embryos. NC arises at the juncture of neural and non-neural ectoderm by the combined action of multiple signals, including BMPs, FGFs, and WNTs [31], with these signals inducing a group of transcription factors with overlapping expression at the NPB. Subsequently, a subset of NPB cells upregulate an additional group of transcription factors in presumptive NC, with these cells delaminating and migrating away from the neural tube shortly thereafter. At 10 hpf, we found that the NPB expression of *msxb*, *pax3a*, *zic2a* and *tfap2a* was indistinguishable between wild-type and *h3f3a^db1092^* embryos (Figure 6a–6d). Neural and otic placode patterning was also normal, as shown by forebrain (*dlx2a*), midbrain (*pax2a*), hindbrain (*egr2b*), and otic (*sox10*) gene expression (Figure 6m; Figure S5). In contrast, the expression of *snai2*, *sox10*, *foxd3*, and *sox9b* was lost or greatly reduced in the presumptive CNC domains of *h3f3a^db1092^* embryos at 11 hpf (Figure 6g–6k), as was *sox10* expression in D123N-H3.3-injected embryos (Figure 6l). However, the NPB-specific expression of *msxb* and *pax3a* remained unaffected at these stages, indicating that the loss of CNC expression was not due to cell loss (Figure 6e, 6f). In particular, the loss of *tfap2a* expression in the later CNC domain (Figure 6j) but not the earlier NPB domain (Figure 6d) of *h3f3a^db1092^* mutants highlights the selective role of H3.3 in CNC gene expression. Interestingly, *h3f3a^db1092^* embryos had no defects in trunk NC formation as assayed by *crestin* and *sox9b* expression at 11.7 hpf (Figure 7), consistent with trunk NC derivatives being unaffected. While it remains unclear why trunk NC is spared, the greater sensitivity of cranial versus trunk NC to defects in H3.3 function correlates with the greatly increased capacity of CNC to generate ectomesenchyme.

**Figure 8:**
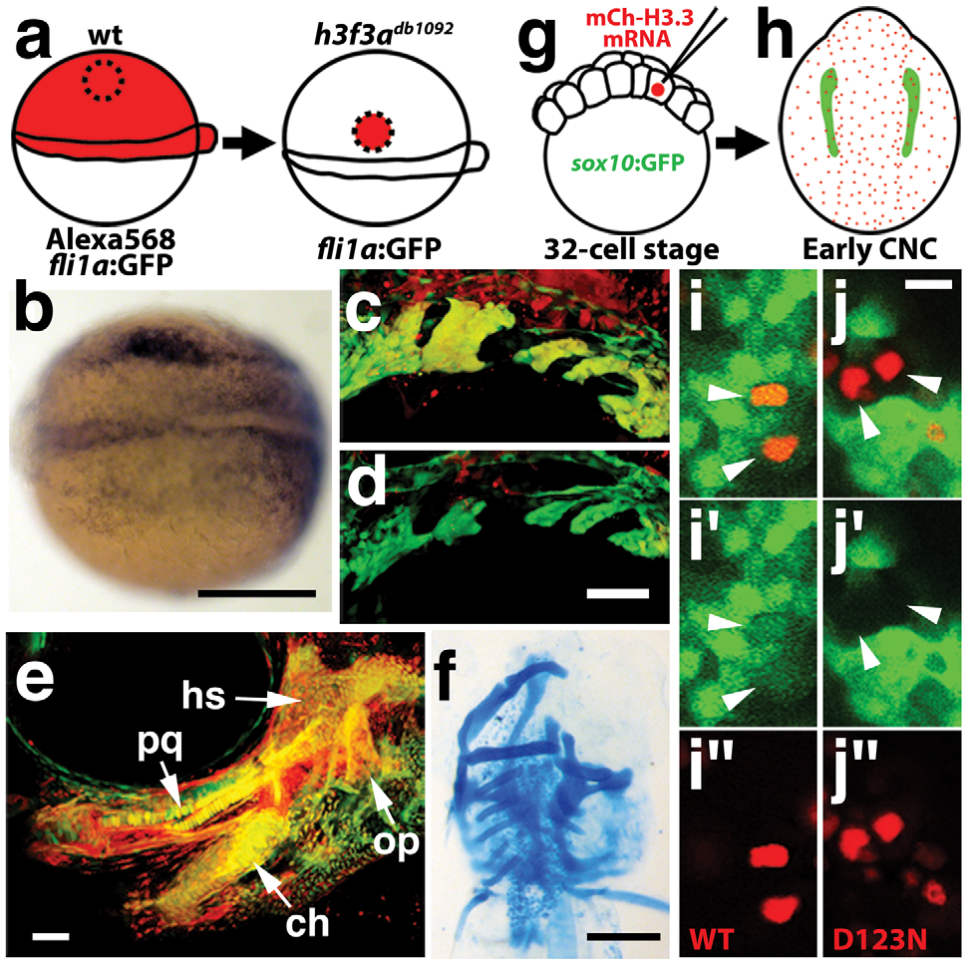
H3.3 function is required tissue- and cell-autonomously for CNC development. a, Wild-type cells were transplanted unilaterally into the CNC precursor domain of *h3f3a^db1092/db1092^* homozygous mutants at 6 hpf. b, Compared to the non-recipient control side (bottom), expression of the early CNC marker *snai2* is restored in the recipient side at 11hpf (top) (n = 17/29 with rescue). c–e, *fli1a*:GFP (green) marks CNC ectomesenchyme of the pharyngeal arches at 30 hpf and facial skeletal elements at 5 dpf. The red fluorescent dye, Alexa568, marks transplanted wild-type cells, whereas both donor and host cells harbor the *fli1a*:GFP transgene. When transplanted into an *h3f3a^db1092/db1092^* homozygous host, wild-type Alexa568+ CNC precursors contribute to pharyngeal arch ectomesenchyme and rescue arch size (c) and form wild-type cartilage and bone (e). In contrast, the non-recipient control side (d) has reduced pharyngeal arch ectomesenchyme. Whereas wild-type donor cells appear yellow due to red Alexa568 and green *fli1a*:GFP, mutant host cells have only *fli1a*:GFP and hence appear green. Rescue of arch size was observed in 8/11 cases. f, Alcian staining at 5 dpf shows that cartilage is restored to half the face in an *h3f3a^db1092/db1092^* larvae that received an unilateral wild-type CNC precursor transplant. Compare the recipient side (left) to the control side that forms little facial cartilage (right). Skeletal rescue was observed in 21/30 cases. g, Individual cells of 32-cell stage *sox10*:GFP embryos were injected with mRNA encoding mCherry-tagged versions of wild-type or D123N H3.3 to generate mosaic mCherry-H3.3 expression at later stages. h, Soon after the appearance of GFP-labeled CNC at approximately 11 hpf, mosaic embryos were assessed for incorporation of mCherry-H3.3-expressing cells (red) into the *sox10*:GFP-positive CNC domain (green). i/i′/i″ and j/j′/j″, Confocal images from *sox10*:GFP embryos with mosaic expression of wild-type (i/i′/i″) and D123N (j/j′/j″) versions of mCherry-H3.3. Cells doubly-positive for wild-type mCherry-H3.3 (red) and GFP (green) (arrowheads) were observed within the CNC domain (7/14 cells over 4 embryos), whereas mutant D123N mCherry-H3.3 cells within the CNC domain failed to up-regulate *sox10*:GFP (arrowheads) (0/18 cells over 4 embryos). Only cells with strong mCherry-H3.3 were used in the analysis. hs: hyosymplectic cartilage, pq: palatoquadrate cartilage, ch: ceratohyal cartilage, op: opercular bone. Scale bars: b, f, 250 µm; c–e, 50 µm; i and j,10 µm.

Consistent with a direct role of H3.3 in CNC development, we also found H3.3 to be required both tissue- and cell-autonomously for CNC specification. Shield-stage transplantation of wild-type CNC precursors into *h3f3a^db1092/db1092^* homozygous mutant hosts fully rescued *snai2* CNC expression, *fli1a*:GFP-positive ectomesenchyme, and head skeleton (Figure 8a–8f). Moreover, mosaic injection of D123N-H3.3 blocked the CNC expression of a *sox10*:GFP transgene in a strictly cell-autonomous manner (Figure 8g–8j).

**Figure 9:**
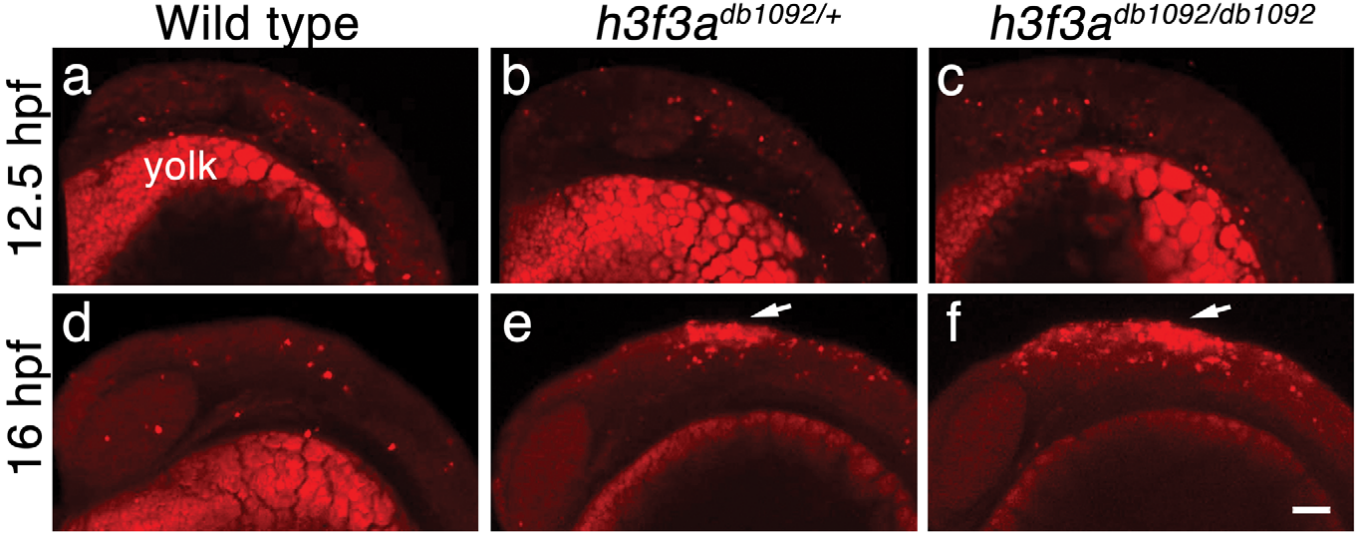
Cell death in *h3f3a^db1092^* embryos. a–c, Lysotracker Red staining marks similar amounts of dying cells in wild-type (n = 2), *h3f3a^db1092/+^* heterozygous (n = 5), and *h3f3a^db1092/db1092^* homozygous (n = 3) embryos at 12.5 hpf. The bright staining in the bottom of each panel is the yolk. d–f, At 16 hpf, increased Lysotracker Red staining (arrows) was evident in the dorsal neural tube of *h3f3a^db1092/+^* heterozygotes (2/2) and *h3f3a^db1092/db1092^* homozygotes (3/3) but not wild types (0/4). These dying cells were located in a similar position to where CNC forms in wild-type embryos. Scale bar = 50 µm.

Although defective at early stages (11 hpf), the CNC expression of *sox10* partially recovered by 16.5 hpf in *h3f3a^db1092^* mutants (Figure 6m), consistent with multiple CNC derivatives being unaffected or only mildly reduced. In contrast, the expression of the ectomesenchyme markers *dlx2a* and *twist1a* was greatly reduced in *h3f3a^db1092^* mutants at 16.5 hpf (Figure 6n, 6o; Figure S5). The loss of ectomesenchyme expression corresponded to regions of *sox10*-expressing CNC that were absent in *h3f3a^db1092^* mutants. CNC cells that normally become ectomesenchyme likely fail to migrate from the neural tube and die, as we observed an increase in *sox10*-positive cells in the dorsal neural tube (Figure 6m) and elevated cell death in this same domain of 16 hpf *h3f3a^db1092^* embryos (Figure 9d–9f). Importantly, increased cell death was not observed in the *h3f3a^db1092^* neural folds at 12.5 hpf (Figure 9a–9c), indicating that the earlier loss of CNC gene expression was not due to cell death.

## Discussion

**Figure 10:**
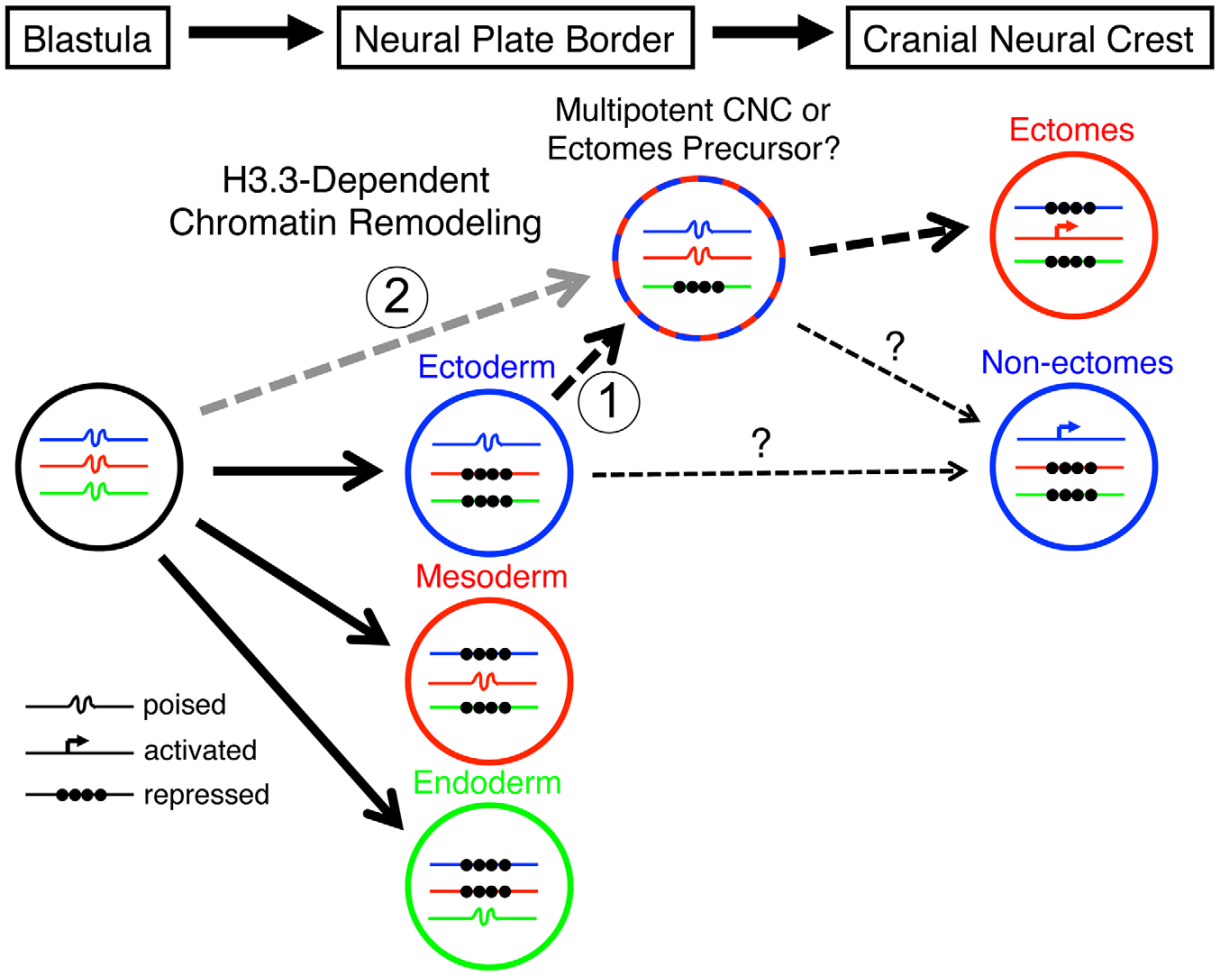
Model for the role of H3.3-dependent histone replacement during CNC development. At the early embryonic blastula stage, cells have a broad potential with cis-regulatory elements for developmental genes existing in a “poised” chromatin state. After gastrulation occurs to form the three major germ layers (ectoderm, mesoderm, and endoderm), genes associated with a particular germ layer are activated or maintained in a poised state, whereas genes for other layers are strongly repressed at the chromatin level. The cranial neural crest (CNC) is unusual in that it is derived from ectoderm yet can give rise to mesoderm-like derivatives such as skeleton. H3.3-dependent histone replacement could thus be required to remodel the enhancers of mesodermal genes needed for ectomesenchymal fates, with the distinctive role of H3.3 in CNC correlating with the need to derepress mesodermal enhancers that have been previously silenced in the ectoderm germ layer (1). Alternatively H3.3 incorporation could act to maintain mesoderm-like potential in the CNC ectoderm from an earlier time in development (2). It also remains unresolved the extent to which ectomesenchyme derivatives (e.g. head skeleton) and non-ectomesenchyme derivatives (e.g. pigment, glia, and neurons) derive from a common multipotent precursor. Hence, the cranial pigment and ectomesenchyme defects of *h3f3a^db1092^* mutants could arise from altered histone replacement in a common multipotent precursor, or alternatively from independent defects in different subsets of heterogeneous CNC with more limited potential.

It is becoming increasingly appreciated that changes in chromatin states are intimately tied to changes in cellular potential. During differentiation of many stem/progenitor cells, combinatorial histone marks associated with the promoters and enhancers of poised developmental genes are progressively resolved to active or inactive states in a lineage-specific manner [7]–[10]. In the germ line, rather than progressive enzymatic modifications of histone marks, the global replacement of existing histones with H3.3 and other variant histones is thought to contribute to a dramatic alteration of lineage potential [17]. The unusual developmental history of ectomesenchyme may explain why it too is uniquely sensitive to defects in the incorporation of H3.3 replacement histones. CNC ectomesenchyme derives from the ectoderm yet expresses a gene repertoire and gives rise to derivatives (e.g. skeleton) in common with the mesoderm. One possibility then is that an H3.3-dependent histone replacement event in the NPB, which by its widespread nature is particularly sensitive to defective H3.3 incorporation, endows ectoderm-derived CNC cells with their exceptionally broad lineage potential, including the ability to generate mesoderm-like derivatives (Figure 10). The requirement for histone replacement (as opposed to enzymatic histone modifications) could reflect a need to overcome a particularly entrenched level of silencing during this fate transition, or alternatively to actively maintain mesoderm-like potential from earlier developmental stages [32]. In contrast, the progressive fate restriction of most other embryonic lineages may depend more on an incremental refinement of chromatin structure, such as the post-translational modification of histone tails and re-positioning of existing nucleosomes, and thus be less sensitive to defective H3.3 incorporation.

The open permissive chromatin structure at poised regulatory regions is characterized by low nucleosome occupancy and high H3.3 levels, although it remains unclear whether H3.3 simply fulfills a neutral replacement function in such areas of high turnover or has a facilitative destabilizing role [33], [34]. An important question is which genes, and in particular which regulatory regions, are targets of histone replacement in the early NPB/CNC. Early CNC genes such as *sox10* and *foxd3*, whose expression is dramatically perturbed in the presence of mutant D123N H3.3, could be direct targets, although it is unclear why their activation would uniquely depend on H3.3. Alternatively or in parallel, the regulatory regions of a larger set of lineage-specific genes, in particular those associated with later ectomesenchyme development, could be primed by H3.3-dependent histone replacement during early CNC specification. Future chromatin immunoprecipitation studies, currently not feasible due to the rarity of CNC precursors in zebrafish embryos, will clearly be critical for determining which types of genes are direct targets of histone replacement during CNC specification.

Another unresolved question is how H3.3 incorporation is specifically targeted to CNC genes/enhancers. Several putative H3.3 chaperones have been identified, including Hira [35], [36], Daxx [37], and Dek [38]. However, effective morpholino-mediated reduction of zebrafish *hira*, *daxx*, or *dek* gene products, either alone or in combination, failed to cause CNC-specific defects (Figure S6). *Hira^−/−^* and *Dek^−/−^* mouse mutants also do not display CNC-specific defects [39], [40], whereas *Daxx^−/−^* embryos die around E8.5 before CNC can be extensively analyzed [41]. Whereas evidence for Hira, Daxx, or Dek mediating H3.3 incorporation in CNC is inconclusive, an intriguing alternative candidate is the chromatin remodeling complex CHD7-PBAF. Losses of CHD7-PBAF components disrupt early CNC specification in a manner similar to mutant D123N H3.3, with reduced expression of early CNC gene expression but not upstream NPB factors [11], [12], and CHD7 genomic localization coincides with H3K4me1 marks which are particularly enriched in H3.3 [34], [42]. It is less clear to what extent CHD7-PBAF, as well as the histone demethylase jmjD2A, share similar requirements with H3.3 for CNC lineage potential. As in *h3f3a^db1092^* mutants, inhibition of CHD7 function in *Xenopus laevis* embryos disrupts craniofacial cartilage development, yet the effects on other NC lineages were not examined. In contrast, depletion of jmjD2A in avians affected development of the CNC-derived ganglia, a structure that is specifically spared in *h3f3a^db1092^* mutants. Whether CHD7-PBAF and jmjD2A act together with H3.3 histone replacement at similar regulatory regions of early CNC genes, or whether these complexes have distinct roles in CNC specification and subsequent lineage diversification, will be fertile areas for future research.

*h3f3a^db1092^* mutants display an initial delay in the expression of all CNC genes examined, which then translates into a more restricted loss of CNC-derived ectomesenchyme. How then does this specific loss of ectomesenchyme relate to the earlier delay in CNC specification? In one model, early-forming CNC cells encounter local environmental cues that promote ectomesenchymal fates [43], with a delay in CNC formation causing cells to miss such cues. Alternatively, the delay in CNC appearance, and its later inability to form the normal range of derivatives, may both result from defects in H3.3-dependent epigenetic remodeling during early CNC stages. Indeed, studies in zebrafish indicate that the future lineage potential of CNC may be determined at very early stages [25]. In addition, the cranial pigment lineage defects of *h3f3a^db1092^* mutants indicate more general roles for H3.3 histone replacement in CNC lineage potential. In contrast to trunk NC [3], the existence of a multipotent precursor for all CNC lineages has yet to be demonstrated in vivo. Evidence in avians suggests that the ectomesenchyme arises from a temporally and spatially distinct subpopulation of CNC from precursors of other derivatives [6], [44], and lineage tracing studies in zebrafish show that CNC cells are largely unipotent at early stages of development (13 hpf) [5], [45]. Hence, despite individual cultured avian CNC cells being able to generate all derivatives [2], the embryonic CNC may be heterogeneous from initial stages. H3.3 histone replacement could therefore be selectively required for ectomesenchyme and pigment cell potential, but not neuroglial potential, within a common multipotent CNC precursor, or alternatively within distinct subpopulations of ectomesenchyme and pigment cell precursors (Figure 10). In the future, techniques to more severely perturb H3.3 histone replacement should help reveal whether the apparent lack of CNC neuroglial and trunk NC defects in *h3f3a^db1092^* mutants reflects a fundamentally different mode of chromatin remodeling in the development of these NC populations, as well as other embryonic cell types, or merely a lower sensitivity to loss of H3.3 function.

## Materials and Methods

### Ethics statement

All animals were handled in strict accordance with good animal practice as defined by the relevant national and/or local animal welfare bodies, and all animal work was approved by the University of Southern California Institutional Animal Care and Use Committee.

### Isolation and genotyping of the *db1092* mutation

For the genetic screen, wild-type AB adult males were mutagenized with ethylnitrosourea and crossed with *fli1a*:GFP females to create *fli1a*:GFP mutant carriers. Using early pressure to inhibit meiosis II, parthenogenic diploid F1 progeny [46] were created from these carriers and analyzed under a fluorescent dissecting microscope for *fli1a*:GFP defects. In order to genotype the *h3f3a^db1092^* mutation, the following primers are used to amplify genomic DNA: 5′ - TTCAACAGGAAGCAAGTGAGG, 3′ – AACCCAATGATCGATGGAAA. As the *h3f3a^db1092^* mutation destroys an EcoRV site, digestion with EcoRV generates a 359 bp product in mutants, and 261 bp and 98 bp fragments in wild types.

### Skeletal staining, in situ hybridization, and immunohistochemistry

Zebrafish were maintained at 28.5°C and staged as described [47]. Acid-free skeletal staining was performed using Alcian Blue for cartilage and Alizarin Red for bone and teeth [48]. In situ hybridizations were performed as described [49]. For in situs, we carefully controlled for potential stage differences caused by mutants, RNA injection, and morpholinos by individually staging embryos by standardized morphological features (e.g. somite number). The following probes were the same as published: *msxb* [50], *pax3a* [51], *zic2a* [52], *snai2* [53], *sox10* [54], *tfap2a* [55], *sox9b* [56], *dlx2a* [57], *pax2a* [58], *egr2b* [59], and *crestin* [60]. *dct*, *xdh*, *foxd3*, *twist1a*, and *h3f3a* probes were made by cDNA amplification from mixed-stage embryos using the following primers, with a T7 RNA polymerase initiation site incorporated into the 3′ primer for probe synthesis (underlined): *dct-5′* - CGTACTGGAACTTTGCGACA, *dct-3′* - GCTAATACGACTCACTATAGGACCAACACGATCAACAGCAG, *xdh-5′* - TGAACACTCTGACGCACCTC, *xdh-3′* - GCTAATACGACTCACTATAGGTGTTGAAGCTCCAGCAACAC, *foxd3-5′* - CGGCATTGGGAATCCATA, *foxd3-3′* – GCTAATACGACTCACTATAGGCAACGAAATGAAATAGAAAGAAGGA, *twist1a-5′* - CAGAGTCTCCGGTGGACAGT, *twist1a-3′* – GCTAATACGACTCACTATAGGGTCTTTTCCTGCAGCGAGTC, and *h3f3a-5′* - TTCAACTTTTAAAGAGAAACACTCAT, *h3f3a-3′* - GCTAATACGACTCACTATAGGCCTTTATCTCTCCATTTATGGTAAAC. The *h3f3a* probe was made against the 3′ untranslated region, which we found to be unique among zebrafish H3.3 genes. *dct*, *xdh*, *foxd3*, and *twist1a* probes gave identical expression patterns to those previously described [61], [62]. For whole-mount immunohistochemistry, the primary antibody HuC (Molecular Probes) was used at 1∶400, a secondary goat anti-mouse Alexa Fluor 568 antibody (Molecular Probes) was used at 1∶200, and embryos were processed as described [63].

With the exception of the experiments in Figure S1 and Figure S5, all embryos were genotyped after image acquisition to confirm the segregation of the observed phenotypes with the *h3f3a^db1092^* mutation. In Figure S1, reductions in the *fli1a*:GFP-positive ectomesenchyme (known to correlate precisely with the presence of the *h3f3a^db1092^* mutation in our other experiments) was used to identify mutant embryos. In Figure S5, the presence of *dlx2a* reductions (also known to correlate with *h3f3a^db1092^* genotype in our other experiments) was used to identify *h3f3a^db1092^* mutants.

### Construction of expression vectors and mRNA injections

Constructs used to generate mRNA were created using a Gateway cloning system (Invitrogen) that has been modified for use in zebrafish [64]. *h3f3a* (wild-type and *db1092*) and H3.2 (cDNA zgc:158629) open reading frames (ORFs) were amplified from embryonic cDNA using primers with attB1 and attB2R 5′ extensions (underlined) for cloning into the pDONR-221 middle entry vector. We also incorporated a consensus Kozak sequence immediately upstream of the start ATG in the forward primer (bold/italic). Primers used include the following: *h3f3a*-attB1 - GGGGACAAGTTTGTACAAAAAAGCAGGCT ***CCACC***ATGGCCCGTACTAAGCAGAC, *h3f3a*-attB2R – GGGGACCACTTTGTACAAGAAAGCTGGGTTTAAGCCCTCTCTCCTCTGAT, H3.2-attB1 - GGGGACAAGTTTGTACAAAAAAGCAGGCT ***CCACC***ATGGCAAGAACCAAGCAGAC, H3.2-attB2R – GGGGACCACTTTGTACAAGAAAGCTGGGTAGCCTTTGGGTTTAAATCAGG. PCR products were cloned into the pDONR-221 vector. Fusion PCR [65] was used to incorporate the D123N mutation into H3.2. In the first step the pDONR-221:H3.2 construct was used as template for PCR with two sets of primer pairs (A and B; C and D), with overlapping primers B and C containing the required mutation (lowercase in primer sequences): AB amplicon: Primer A (upstream of pDONR-221 integration site) - CAAATTGATGAGCAATGCTTTTT, Primer B – TGGATGTtCTTGGGCATGAT; CD amplicon: Primer C – CATGCCCAAGaACATCCAG, Primer D (downstream of pDONR-221 integration site) – GAGCTGCCAGGAAACAGCTA. In the second step these two PCR products were combined and used as template for PCR with the H3.2-attB1 and H3.2-attB2R pDONR-221 cloning primers above, followed by cloning into pDONR-221. The fusion PCR technique was also used to generate N-terminal mCherry fusions for *h3f3a* and H3.2 constructs. Primers were designed to amplify the mCherry ORF excluding the stop codon (primers A and B) and the *h3f3a*/H3.2 ORF (primers C and D), with overlapping primers B and C encoding the linker sequence GSRPVAT (used previously for YFP-H3.3 N-terminal fusions [66] ; linker sequence in lowercase below) and primers A and D having attB1 and attB2R 5′ extensions (underlined), respectively: mCherry-A - GGGGACAAGTTTGTACAAAAAAGCAGGCT ***CCACC***ATGGTGAGCAAGGGCGAGG, mCherry-B - tgtagccacaggtctagatccCTTGTACAGCTCGTCCATGC, *h3f3a*-C - ggatctagacctgtggctacaATGGCCCGTACTAAGCAGAC, *h3f3a*-D - *h3f3a*-attB2R (above), H3.2-C - ggatctagacctgtggctacaATGGCAAGAACCAAGCAGAC, H3.2-D - H3.2-attB2R (above). PCR with AB and CD primer pairs were performed using the appropriate plasmid template (pME-mCherry, pDONR-221:*h3f3a* or pDONR-221:H3.2) followed by gel purification of the products. In the second step these two PCR products were combined (mCherry product AB with product CD from *h3f3a* or H3.2) and used as template for PCR AD with the mCherry-A and *h3f3a*-attB2R/H3.2-attB2R cloning primers above, followed by cloning into pDONR-221. All pDONR-221 middle entry clones were checked by sequencing, before being assembled with a 5′ entry vector (p5E-CMV/SP6 containing the Sp6 RNA polymerase promoter) and a 3′ entry vector (p3E-polyA containing the SV40 late polyA signal) within the pDestTol2pA2 destination vector using LR clonase II Plus (Invitrogen), as per manufacturer's instructions. In order to generate capped mRNA from these constructs, plasmid minipreps were first linearized using an appropriate restriction enzyme(s) (pDONR-221:*h3f3a* – BglII, pDONR-221:H3.2 - XbaI, pDONR-221:mCherry-*h3f3a* - SpeI and XhoI, pDONR-221:mCherry-H3.2 – SpeI and XhoI) and then used as template for in vitro transcription using the Sp6 Message Machine kit (Ambion). Zebrafish embryos were injected at the one-cell stage with capped mRNA at a concentration of 900 ng/µl (*h3f3a*, H3.2, mCherry-*h3f3a* and mCherry-H3.2) or 450 ng/ul (*h3f3a* mRNA rescue of *h3f3a^db1092^* mutants). Alternatively individual cells of 32-cell stage embryos were injected with mRNA encoding mCherry-*h3f3a* (900 ng/µl) to generate mosaic expression at later stages.

For the generation of constructs expressing FLAG-HA-tagged H3.3 in HEK 293T cells, the ORF of *h3f3a* was first amplified from wild-type and *db1092* embryonic cDNA using forward and reverse primers with 5′ extensions containing EcoR1 and BamHI sites, respectively: 5′ – GCCGACGGAATTCAGATGGCCCGTACTAAGCAGAC, 3′ - GCCTAGTGGAT CCATTAAGCCCTCTCTCCTCTGAT. Using these restriction sites the PCR products were then cloned between EcoRI and BamHI sites downstream and in-frame with a Kozak-FLAG-HA sequence within a modified pIRESneo vector (Clontech).

The *pax3a*:GFP line will be described in detail elsewhere. Briefly, the *pax3a* enhancer was identified by sequence conservation, then amplified from *Fugu rubripes* genomic DNA and cloned into the Tol2 transgenesis vector pGreenE (unpublished) using BP clonase (Invitrogen). Constructs were injected with transposase mRNA into one-cell-stage embryos and germline transgenic founders were identified.

### Nucleosome incorporation assay

HEK 293T cells were transfected with plasmids expressing FLAG-HA-tagged wild-type H3.3 (pIRES-FLAG-HA-H3.3), or mutant D123N H3.3 (pIRES-FLAG-HA-D123N-H3.3). To purify the whole cell nuclear extract, cultured cells were washed with PBS and lysed in IP lysis buffer (50 mM HEPES-KOH pH 7.6, 140 mM NaCl, 1 mM EDTA, and 10% Triton X-100) followed by western blotting using FLAG antibody (Sigma). To purify mononucleosomes, cell nuclei were digested with micrococcal nuclease (0.6 U, Sigma) as previously described [67] after expression of FLAG-tagged wild-type or D123N H3.3. Purification of mononucleosomes was confirmed by 2% agarose gel electrophoresis. For immunoprecipitation of FLAG-H3.3-containing mononucleosomes, M2-agarose beads (Sigma) were used followed by overnight incubation. Beads were washed five times with BC300 and mononucleosomes were electrophoretically analyzed on a 15% SDS polyacrylamide gel.

### Co-immunoprecipitation

HEK 293T cells were transfected with pIRES-FLAG-HA-H3.3 or pIRES-FLAG-HA-D123N-H3.3. Cells were harvested 2 days after transfection and nuclear fraction was done as previously described [68] . 1 mg of nuclear fraction was incubated with 10 µl Flag Agarose (Sigma) for 4 hr at 4°C in nuclear extraction buffer (20 mM HEPES-KOH (pH 8.0), 0.6 M KCl, 1.5 mM MgCl_2_, 0.2 mM EDTA, 25% (vol/vol) glycerol, 1 mM DTT, 0.2 mM PMSF). This was followed by three washes in IP buffer and elution with 0.2 mg/ml Flag peptide (Sigma). Eluates were subjected to SDS-PAGE and western blot analysis and recombinant octomer was used as a control for western blot. Antibodies used for western blots were as follows: Flag (Sigma), H3 (Abcam), and H4 (Abcam).

### Antisense morpholino oligonucleotides

The following antisense morpholino oligonucleotides were designed to block splicing of exon/intron boundaries in the transcripts of target zebrafish genes (designed by and ordered from Gene Tools, LLC): *h3f3a* exon 3/intron 3/4 – ACAATATAATCTCACCTGAAGAGCG, *hira* exon 1/intron 1/2 – GTCGGTTACTGCTCTCACCATTGTG, *daxx* exon 3/intron 3/4 – ATTACTGTAAACAAGCATACCTCAT, *dek* exon 3/intron 3/4 - ATCTCAAAACAACGCCTTACAGCCC. One-cell stage embryos were injected with 3 nl of morpholino (MO) at 400 µM. RT-PCR was used to determine morpholino efficacy. Whole RNA was prepared from twenty 10 hpf embryos for each treatment (morpholino-injected and uninjected) using the RNAqeous -4PCR kit (Ambion), followed by cDNA synthesis using the RETROscript kit (Ambion). cDNA was used as template for PCR at 50 ng/µl using primers within exons flanking the targeted exon/intron boundary: *h3f3a* exon 3 forward - TCAGCGTCTGGTCAGAGAAA, *h3f3a* exon 4 reverse - TGCTTTACAAAATGACTCCAATG, *hira* exon 1 forward - AAGCTCTTGAAGCCGAGTTG, *hira* exon 2 reverse - TTGGCCACCTGTAGCAAACT, *daxx* exon 3 forward - CACGTGCTGAAAGTGGAGAA, *daxx* exon 4 reverse - TCCACAGACGTCAGAAAGGTC, *dek* exon 3 forward - CCGAAGATTGAGAGCAAAGG, *dek* exon 4 reverse - ATCCATTAAAGAGCCGCAGA.

### Scoring the severity of the *h3f3a^db1092^* phenotype

Alcian Blue-stained 4 and 5 dpf larval head skeletons were scored blind for loss of cartilage elements using the following system: Grade 0, unaffected; Grade 1, truncation of dorsal hyosymplectic and palatoquadrate cartilages; Grade 2, loss of hyosymplectic and palatoquadrate (except pterygoid process), Meckel's and ceratohyal cartilage reduced; Grade 3, loss of Meckel's, ceratohyal and pterygoid; Grade 4, ethmoid plate truncated/lost, trabeculae remain; Grade 5, all CNC-derived cartilages absent.

### NC transplantation

Unilateral tissue transplants were performed as described, with the non-recipient side acting as an internal control [69]. Briefly, one-cell-stage donor *fli1a*:GFP embryos were injected with Alexa 568 dye (Molecular Probes) and cells were moved to the CNC precursor domain of *fli1a*:GFP hosts at 6 hpf.

### Lysotracker staining for cell death

Live embryos were manually removed from their chorions and incubated with Lysotracker Red (Invitrogen) diluted to 5 µM in embryo medium (EM). Embryos were then incubated for 30–45 minutes in darkness. After 4–5 rinses with EM, embryos were fixed overnight with 4% PFA in PBS at 4°C. Embryos were then rinsed once with PBT before being dehydrated in a methanol/PBT series to remove background, and then stored at −20°C. Samples were subsequently rehydrated, followed by two washes in PBT, before being imaged by confocal microscopy.

### Equipment and settings

Live embryos, whole-mount skeletal preparations, and in situ hybridization embryos were photographed with a Leica MZ16 stereomicroscope using a Canon PowerShotS80 digital camera and CameraWindow software. Fluorescent images were captured on a Zeiss LSM5 Pascal upright confocal microscope using excitation from Argon 488 and HeNe 543 lasers. Using Zeiss LSM software, a Z-stack of approximately 40 µM was captured for *fli1a*:GFP images and then digitally flattened into a single projection. For the images of fluorescently labeled histones, individual Z-sections from time-lapse movies were used. Next, all files were loaded into Adobe Photoshop CS2 and adjusted for levels and brightness and contrast. Care was taken to apply identical adjustments to images from the same set of experiments, and levels adjustments were limited to avoid removing information from the image.

## Supporting Information

Figure S1 NC derivatives in *h3f3a^db1092^* mutants. a–d, Live views at 32 hpf (a, b) and 54 hpf (c, d) show reductions of head melanocytes (black) in *h3f3a^db1092/db1092^* homozygotes (n = 22/28). The melanocytes of the eye and trunk were never affected. Cranial xanthophores (yellow), most clearly seen at the anterior limit of the head (arrowheads), were also largely unaffected. e, f, In confocal projections of *fli1a*:GFP embryos at 36 hpf, HuC antibody staining (red) labels neurons of the cranial ganglia – from left to right the trigeminal, anterior lateral line, auditory, and posterior lateral line – which are unaffected in *h3f3a^db1092/db1092^* homozygotes (n = 8). In the merged images, *fli1a*:GFP (green) shows a reduction of CNC-derived ectomesenchyme in the mutant. Scale bars: a–d, 250 µm; e & f, 50 µm.
(TIF)

Figure S2 D123N H3.3 fails to localize to condensed chromosomes within NPB cells. Confocal images of 13 hpf embryos harboring the H2A.F/Z:GFP and NPB-specific *pax3a*:GFP transgenes and injected with wild-type or D123N versions of mCherry(m)H3.3 fusion proteins. a–d, GFP fluorescence of cells within the *pax3a*:GFP-labeled NPB domain. Whereas most cells are in interphase, the few cells in metaphase/anaphase (arrowheads in merged images: a″–d″) exhibit both GFP-labeled condensed chromosomes (H2A.F/Z:GFP) and more diffuse lower-level cytoplasmic GFP (*pax3a*:GFP). a′–b′, Wild-type mCherry-H3.3 localizes within the condensed chromosomes of 14/14 metaphase/anaphase cells. c′–d′, D123N mCherry-H3.3 fails to localize within condensed chromosomes and instead appears diffuse throughout *pax3a*:GFP-positive NPB cells after nuclear envelope breakdown. (0/14 cells exhibit chromosomal localization during metaphase/anaphase). Scale bar = 10 µm.
(TIF)

Figure S3 D123N H3.3 protein remains stable during mitosis. Time course of confocal images from H2A.F/Z:GFP embryos expressing D123N mCherry(m)H3.3 fusion protein, showing a cell (arrowhead in merged image a″) progressing through the stages of mitosis including metaphase (a/a′/a″ and b/b′/b″), anaphase (c/c′/c″ and d/d′/d″), telophase (e/e′/e″) and the eventual establishment of two new daughter cells (arrowheads, f/f′/f″). a–d, H2A.F/Z:GFP localizes to condensed chromosomes during both metaphase and anaphase. a′–d′, In contrast, D123N mCherry-H3.3 fails to co-localize with H2A.F/Z:GFP and appears as a weak diffuse signal throughout the cell(s) after nuclear envelope breakdown. e/e′/e″, H2A.F/Z:GFP and D123N mCherry-H3.3 subsequently become co-localized during the re-establishment of the nuclear membranes during telophase. f/f′/f″, Nuclear co-localization continues into interphase in both daughter cells. The rapid re-appearance of strong nuclear mCherry-H3.3 signal in telophase (16/16 cells over 2 embryos) confirms that the low-level diffuse D123N mCherry-H3.3 signal observed during metaphase/anaphase results from a failure to localize to condensed chromosomes rather than protein degradation. Scale bar = 10 µm.
(TIF)

Figure S4 Localization of wild-type mCherry-H3.3 within metaphase/anaphase cells of *h3f3a^db1092/db1092^* embryos. Confocal images from wild-type and *h3f3a^db1092/db1092^* homozygous embryos harboring the H2A.F/Z:GFP transgene and injected with mRNA encoding wild-type mCherry(m)H3.3 fusion protein. Merged images show that wild-type mH3.3 protein co-localizes with H2A.F/Z:GFP in the chromosomes of metaphase/anaphase cells (metaphase cells shown: arrowheads) in wild-type and *h3f3a^db1092/db1092^* homozygotes (mutant, 21/21 cells in 3 embryos; wild-type, 15/15 cells in 3 embryos). Detailed analysis of fluorescence levels revealed no significant differences in the distribution of wild-type mH3.3 fluorescence between wild types and mutants. Scale bar = 10 µm.
(TIF)

Figure S5 *h3f3a^db1092^* embryos lack CNC ectomesenchyme but have normal neural patterning. a, In wild-type embryos at 15.5 hpf, expression of *dlx2a* (blue) marks a subset of the forebrain (FB) and three streams of migrating CNC-derived ectomesenchyme (1–3). In red, *pax2a* expression marks the mid-hindbrain boundary (MHB) and *egr2b* expression marks rhombomeres 3 and 5 (R3 and R5) of the hindbrain. b, c, *dlx2a*-positive ectomesenchyme is reduced (n = 18/18) in *h3f3a^db1092/+^* heterozygous embryos and completely lost (n = 3/9) or greatly reduced (n = 6/9) in *h3f3a^db1092^* homozygotes at similar stages. Neural patterning was never affected in *h3f3a^db1092^* heterozygotes and homozygotes. Scale bar = 100 µm.
(TIF)

Figure S6 Antisense morpholino targeting of H3.3 chaperones. a, Zebrafish have one predicted copy each of *hira*, *daxx*, and *dek* genes in their genomes. Antisense morpholino oligonucleotides were designed to inhibit splicing at specific exon/intron boundaries (green arrowheads) within *hira* (exon 1/intron 1–2), *daxx* (exon 3/intron 3–4), and *dek* (exon 3/intron 3–4) transcripts. Morpholinos were injected into one-cell-stage zebrafish embryos at 400 µM. b–d, Morpholino efficacy was demonstrated by PCR amplification between exons flanking targeted splice junctions from 10 hpf cDNA from 20 pooled embryos (position of primers shown as red arrows in a). Morpholino-treated samples exhibited a significant decrease in PCR product representing spliced transcript (b, *hira*, 96 bp; c, *daxx*, 143 bp; d, *dek*, 169 bp) and a concomitant increase in un-spliced PCR product (b, *hira*, 637 bp; d, *dek*, 247 bp) or an alternative spliced transcript (c, *daxx*, 169 bp: utilization of cryptic splice donor site 26 nucleotides into adjacent intron, predicted to result in frameshift and early termination). e–j, Wholemount in situ hybridization for *sox10* at 11 hpf. Morpholinos against *hira* (f), *daxx* (h) and *dek* (j) have no effect on early *sox10* expression within CNC cells when compared to uninjected controls (e, g, i) (n≥9 for each). k–p, 5 dpf larval head skeletons stained with Alcian blue (cartilage) and Alizarin red (bone and teeth). Craniofacial development is unaffected in surviving *hira* (l), *daxx* (n) and *dek* (p) morpholino-treated individuals and uninjected controls (k, m, o) (n≥35 for each). *hira* morpholino-injected embryos did exhibit a high level of death after 24 hpf but prior to 5 dpf (morpholino injected, 55.1%; uninjected, 0%) and a curved/kinked tail phenotype in surviving 5 dpf larvae (morpholino injected, 48.6%; uninjected, 0%). q–t, Compared to uninjected controls (q & s), embryos injected with a combination of all three morpholinos at 200 µM had no defects in CNC *sox10* expression (r) (n≥9 for each) or development of the larval head skeleton (t) (n≥66 for each). un, uninjected; MO, morpholino. Scale bars = 250 µm.
(TIF)
