# Supplementary figures and images for: An Essential Role of Variant Histone H3.3 for Ectomesenchyme Potential of the Cranial Neural Crest
Source: PLoS Genet. 2012 Sep 20;8(9):e1002938. doi: 10.1371/journal.pgen.1002938 (PMC3447937; doi:10.1371/journal.pgen.1002938)

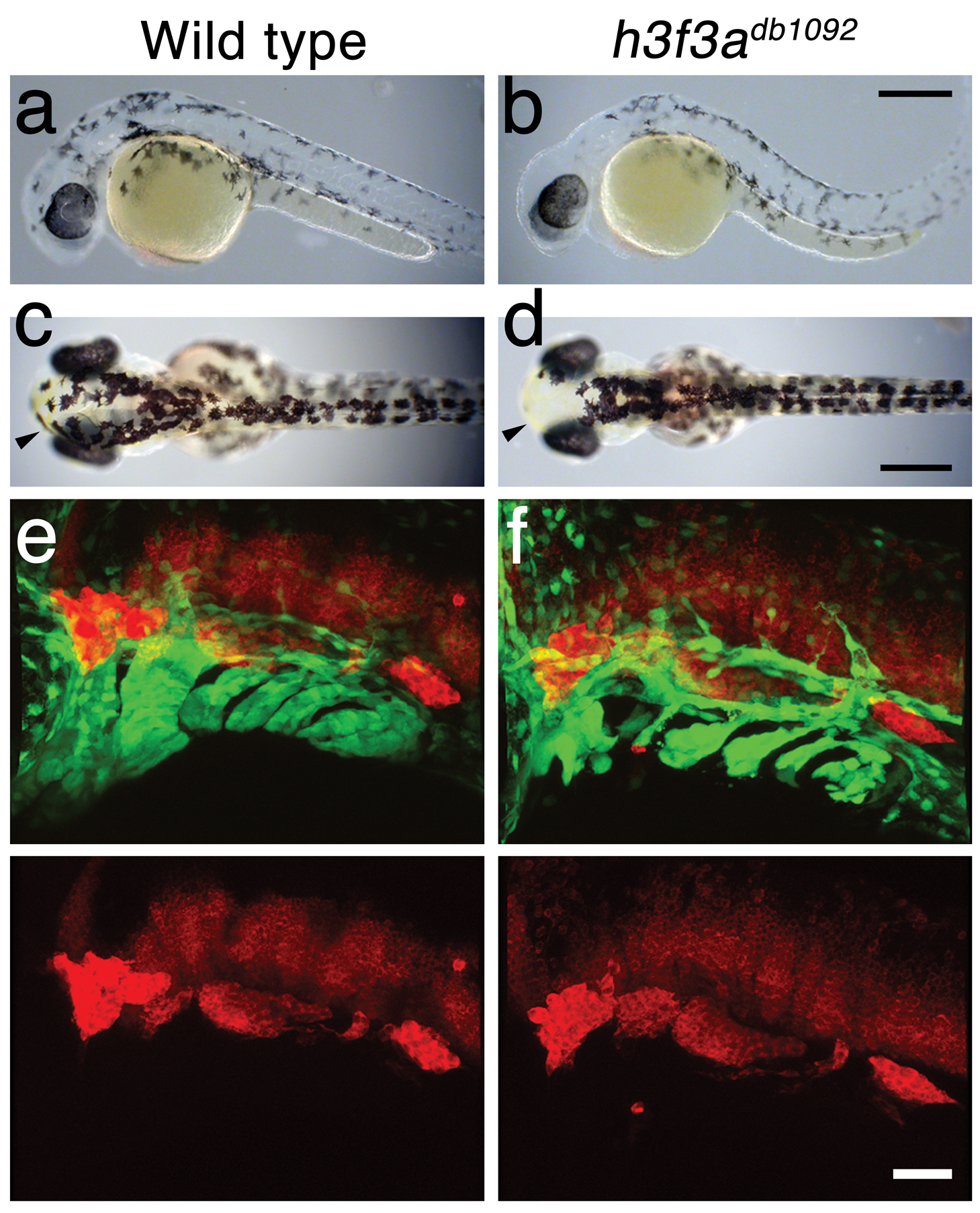

Supplement: Figure S1 — NC derivatives in h3f3adb1092 mutants. a–d, Live views at 32 hpf (a, b) and 54 hpf (c, d) show reductions of head melanocytes (black) in h3f3adb1092/db1092 homozygotes (n = 22/28). The melanocytes of the eye and trunk were never affected. Cranial xanthophores (yellow), most clearly seen at the anterior limit of the head (arrowheads), were also largely unaffected. e, f, In confocal projections of fli1a:GFP embryos at 36 hpf, HuC antibody staining (red) labels neurons of the cranial ganglia – from left to right the trigeminal, anterior lateral line, auditory, and posterior lateral line – which are unaffected in h3f3adb1092/db1092 homozygotes (n = 8). In the merged images, fli1a:GFP (green) shows a reduction of CNC-derived ectomesenchyme in the mutant. Scale bars: a–d, 250 µm; e & f, 50 µm. (TIF) [file pgen.1002938.s001.tif]

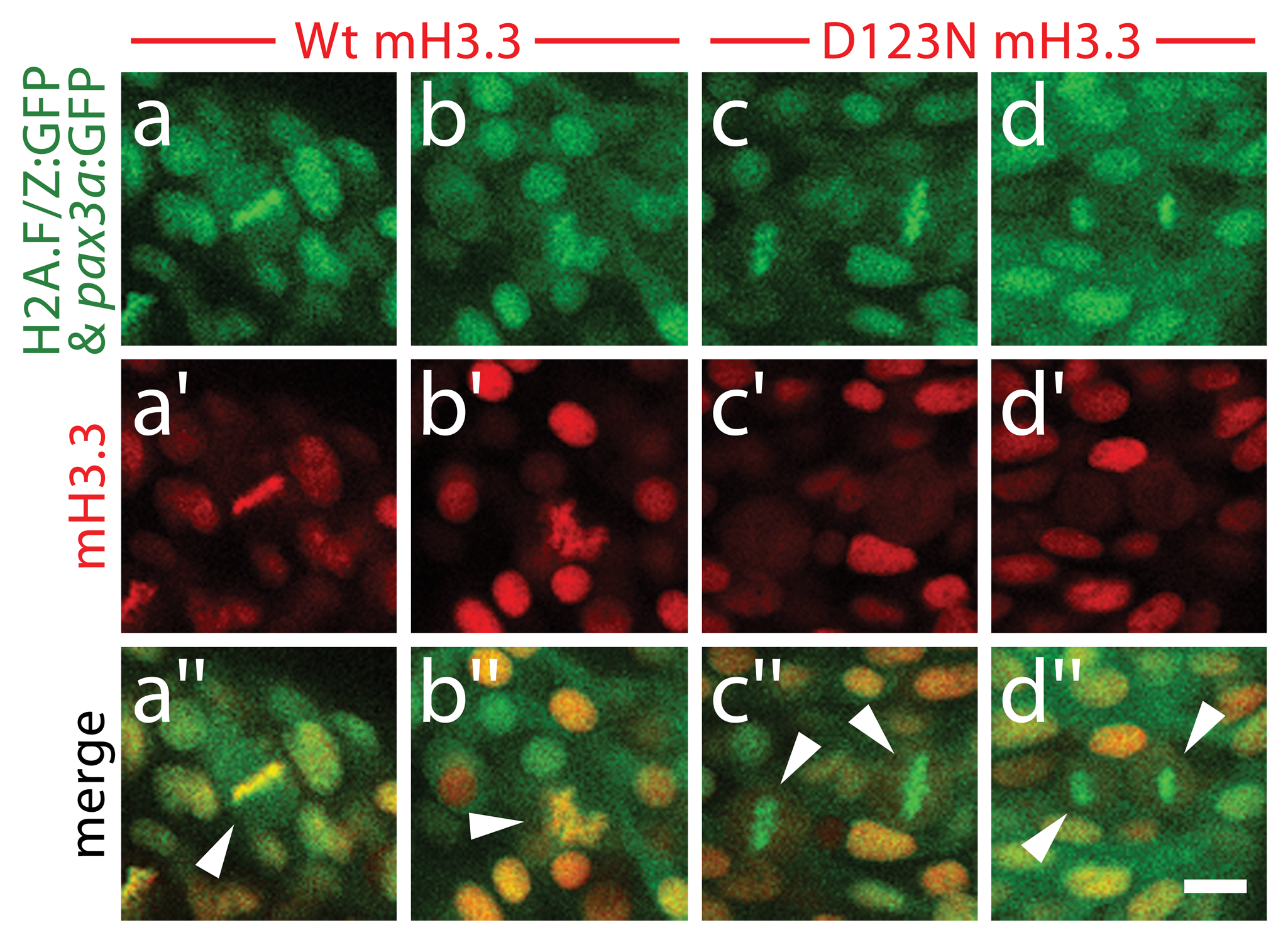

Supplement: Figure S2 — D123N H3.3 fails to localize to condensed chromosomes within NPB cells. Confocal images of 13 hpf embryos harboring the H2A.F/Z:GFP and NPB-specific pax3a:GFP transgenes and injected with wild-type or D123N versions of mCherry(m)H3.3 fusion proteins. a–d, GFP fluorescence of cells within the pax3a:GFP-labeled NPB domain. Whereas most cells are in interphase, the few cells in metaphase/anaphase (arrowheads in merged images: a″–d″) exhibit both GFP-labeled condensed chromosomes (H2A.F/Z:GFP) and more diffuse lower-level cytoplasmic GFP (pax3a:GFP). a′–b′, Wild-type mCherry-H3.3 localizes within the condensed chromosomes of 14/14 metaphase/anaphase cells. c′–d′, D123N mCherry-H3.3 fails to localize within condensed chromosomes and instead appears diffuse throughout pax3a:GFP-positive NPB cells after nuclear envelope breakdown. (0/14 cells exhibit chromosomal localization during metaphase/anaphase). Scale bar = 10 µm. (TIF) [file pgen.1002938.s002.tif]

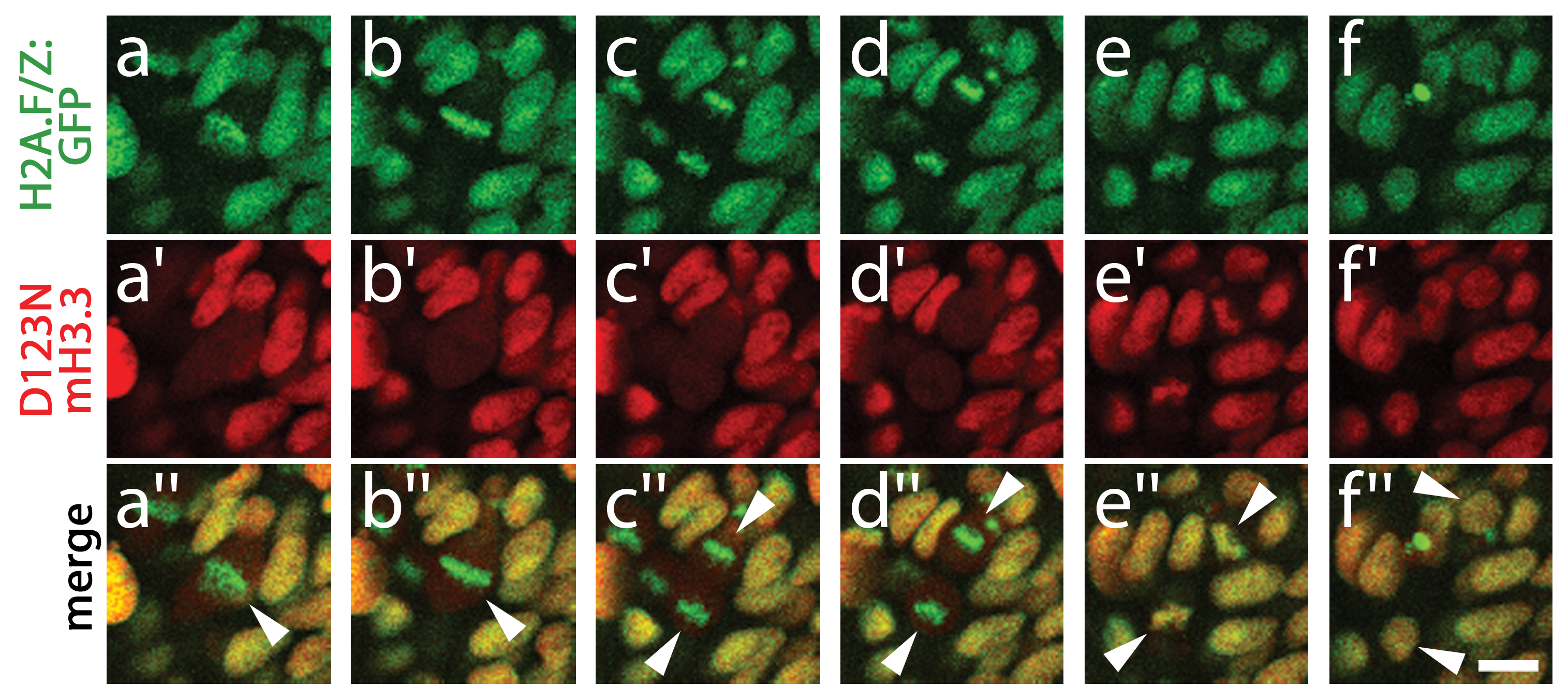

Supplement: Figure S3 — D123N H3.3 protein remains stable during mitosis. Time course of confocal images from H2A.F/Z:GFP embryos expressing D123N mCherry(m)H3.3 fusion protein, showing a cell (arrowhead in merged image a″) progressing through the stages of mitosis including metaphase (a/a′/a″ and b/b′/b″), anaphase (c/c′/c″ and d/d′/d″), telophase (e/e′/e″) and the eventual establishment of two new daughter cells (arrowheads, f/f′/f″). a–d, H2A.F/Z:GFP localizes to condensed chromosomes during both metaphase and anaphase. a′–d′, In contrast, D123N mCherry-H3.3 fails to co-localize with H2A.F/Z:GFP and appears as a weak diffuse signal throughout the cell(s) after nuclear envelope breakdown. e/e′/e″, H2A.F/Z:GFP and D123N mCherry-H3.3 subsequently become co-localized during the re-establishment of the nuclear membranes during telophase. f/f′/f″, Nuclear co-localization continues into interphase in both daughter cells. The rapid re-appearance of strong nuclear mCherry-H3.3 signal in telophase (16/16 cells over 2 embryos) confirms that the low-level diffuse D123N mCherry-H3.3 signal observed during metaphase/anaphase results from a failure to localize to condensed chromosomes rather than protein degradation. Scale bar = 10 µm. (TIF) [file pgen.1002938.s003.tif]

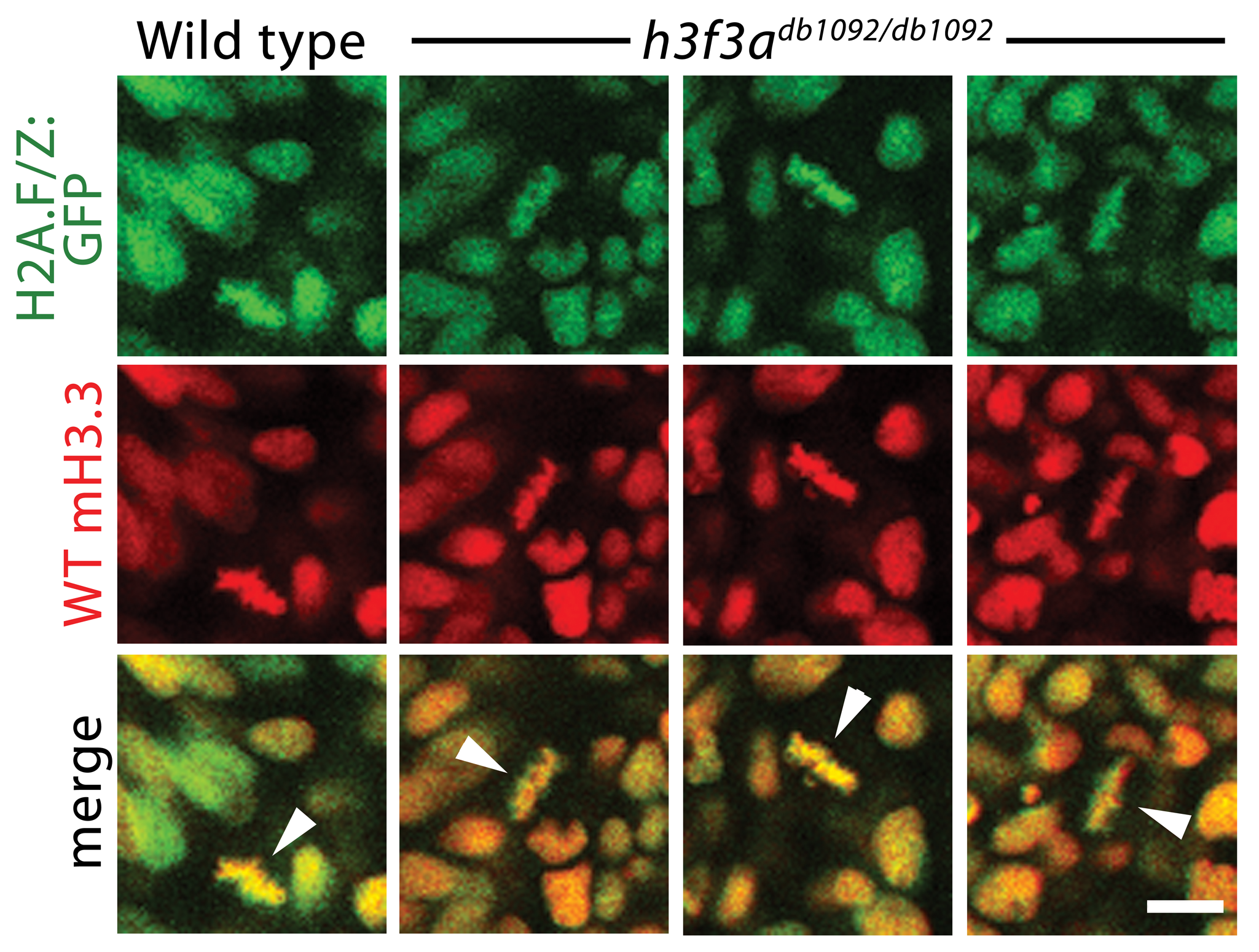

Supplement: Figure S4 — Localization of wild-type mCherry-H3.3 within metaphase/anaphase cells of h3f3adb1092/db1092 embryos. Confocal images from wild-type and h3f3adb1092/db1092 homozygous embryos harboring the H2A.F/Z:GFP transgene and injected with mRNA encoding wild-type mCherry(m)H3.3 fusion protein. Merged images show that wild-type mH3.3 protein co-localizes with H2A.F/Z:GFP in the chromosomes of metaphase/anaphase cells (metaphase cells shown: arrowheads) in wild-type and h3f3adb1092/db1092 homozygotes (mutant, 21/21 cells in 3 embryos; wild-type, 15/15 cells in 3 embryos). Detailed analysis of fluorescence levels revealed no significant differences in the distribution of wild-type mH3.3 fluorescence between wild types and mutants. Scale bar = 10 µm. (TIF) [file pgen.1002938.s004.tif]

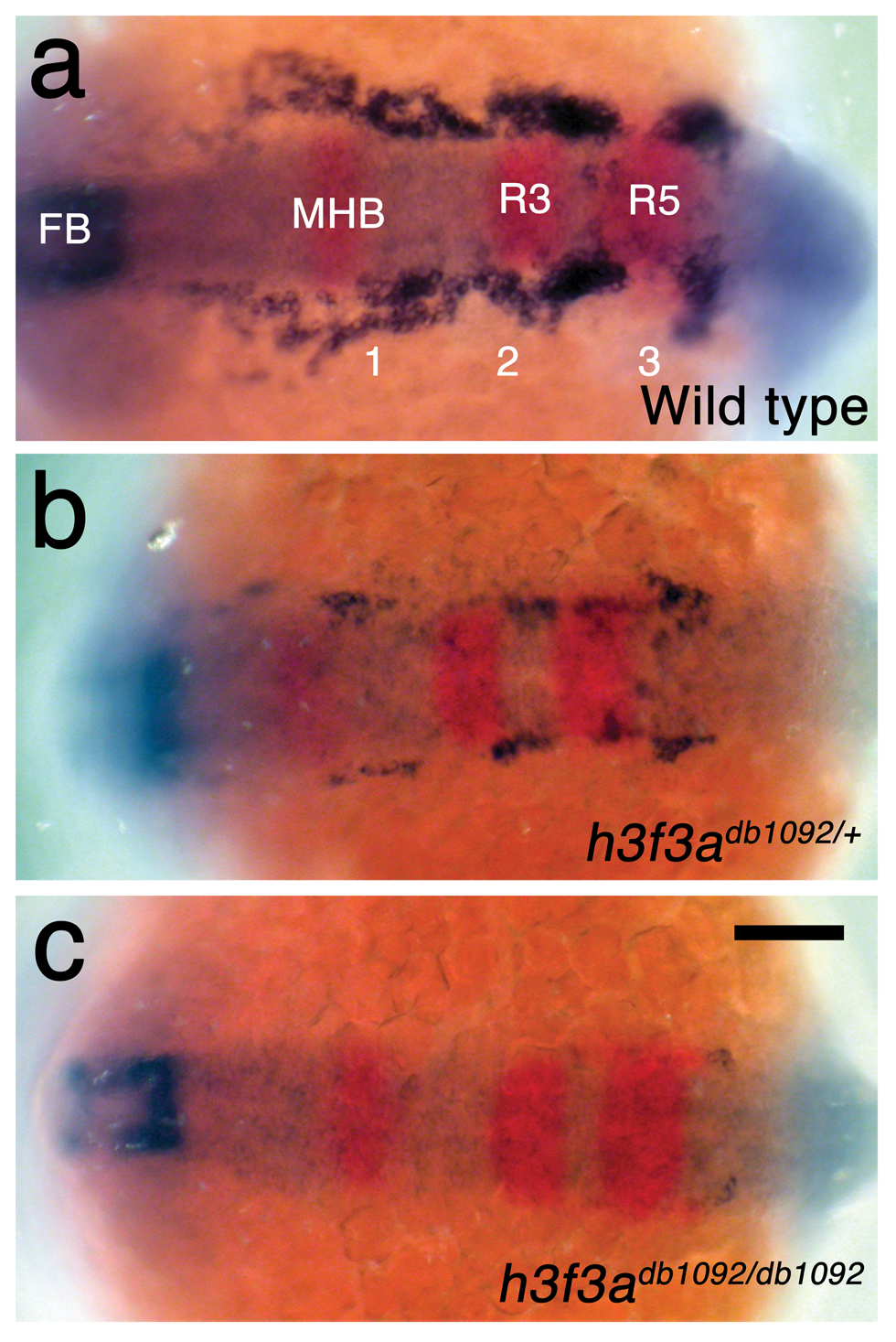

Supplement: Figure S5 — h3f3adb1092 embryos lack CNC ectomesenchyme but have normal neural patterning. a, In wild-type embryos at 15.5 hpf, expression of dlx2a (blue) marks a subset of the forebrain (FB) and three streams of migrating CNC-derived ectomesenchyme (1–3). In red, pax2a expression marks the mid-hindbrain boundary (MHB) and egr2b expression marks rhombomeres 3 and 5 (R3 and R5) of the hindbrain. b, c, dlx2a-positive ectomesenchyme is reduced (n = 18/18) in h3f3adb1092/+ heterozygous embryos and completely lost (n = 3/9) or greatly reduced (n = 6/9) in h3f3adb1092 homozygotes at similar stages. Neural patterning was never affected in h3f3adb1092 heterozygotes and homozygotes. Scale bar = 100 µm. (TIF) [file pgen.1002938.s005.tif]

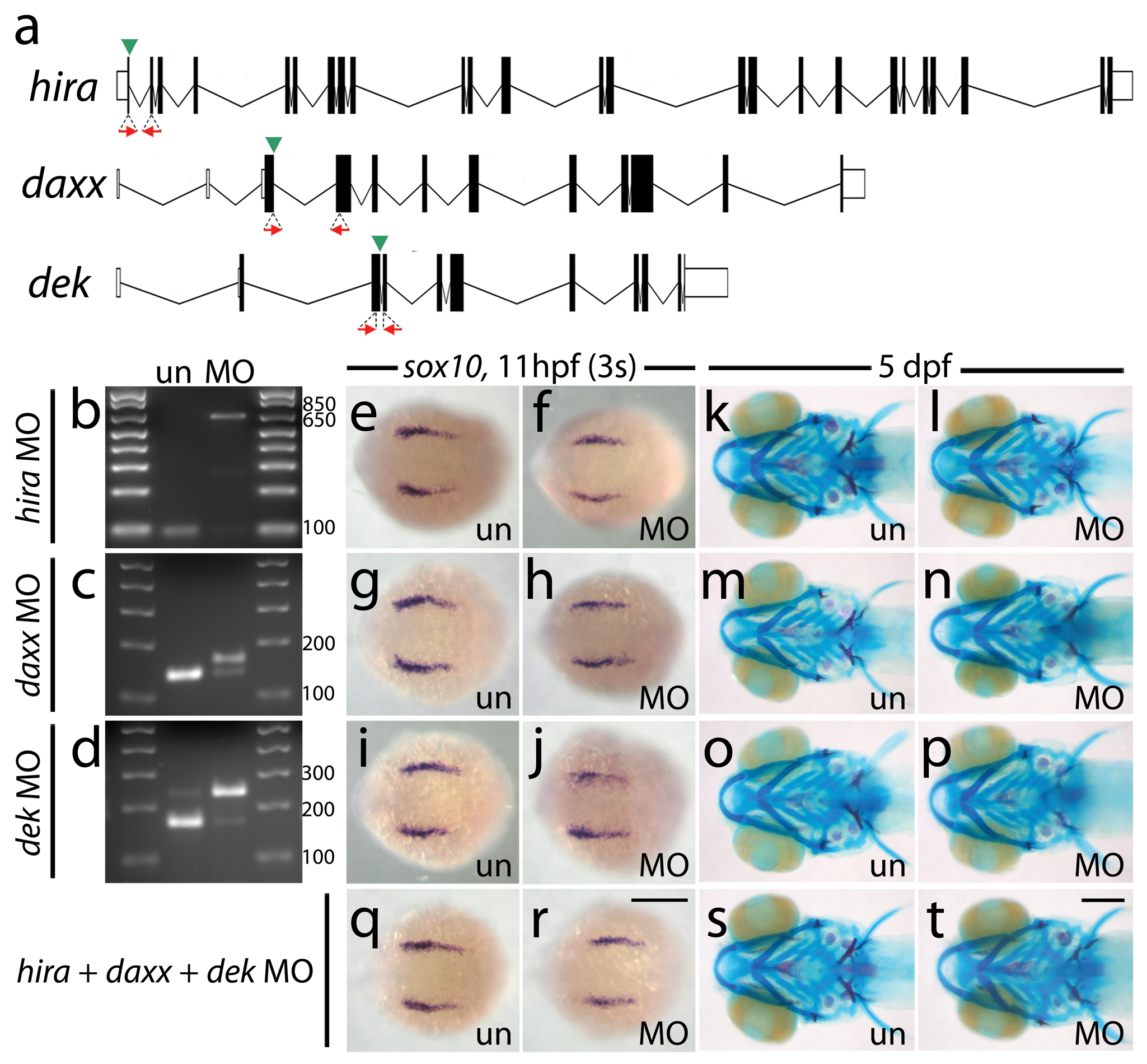

Supplement: Figure S6 — Antisense morpholino targeting of H3.3 chaperones. a, Zebrafish have one predicted copy each of hira, daxx, and dek genes in their genomes. Antisense morpholino oligonucleotides were designed to inhibit splicing at specific exon/intron boundaries (green arrowheads) within hira (exon 1/intron 1–2), daxx (exon 3/intron 3–4), and dek (exon 3/intron 3–4) transcripts. Morpholinos were injected into one-cell-stage zebrafish embryos at 400 µM. b–d, Morpholino efficacy was demonstrated by PCR amplification between exons flanking targeted splice junctions from 10 hpf cDNA from 20 pooled embryos (position of primers shown as red arrows in a). Morpholino-treated samples exhibited a significant decrease in PCR product representing spliced transcript (b, hira, 96 bp; c, daxx, 143 bp; d, dek, 169 bp) and a concomitant increase in un-spliced PCR product (b, hira, 637 bp; d, dek, 247 bp) or an alternative spliced transcript (c, daxx, 169 bp: utilization of cryptic splice donor site 26 nucleotides into adjacent intron, predicted to result in frameshift and early termination). e–j, Wholemount in situ hybridization for sox10 at 11 hpf. Morpholinos against hira (f), daxx (h) and dek (j) have no effect on early sox10 expression within CNC cells when compared to uninjected controls (e, g, i) (n≥9 for each). k–p, 5 dpf larval head skeletons stained with Alcian blue (cartilage) and Alizarin red (bone and teeth). Craniofacial development is unaffected in surviving hira (l), daxx (n) and dek (p) morpholino-treated individuals and uninjected controls (k, m, o) (n≥35 for each). hira morpholino-injected embryos did exhibit a high level of death after 24 hpf but prior to 5 dpf (morpholino injected, 55.1%; uninjected, 0%) and a curved/kinked tail phenotype in surviving 5 dpf larvae (morpholino injected, 48.6%; uninjected, 0%). q–t, Compared to uninjected controls (q & s), embryos injected with a combination of all three morpholinos at 200 µM had no defects in CNC sox10 expression (r) (n≥9 for eac [file pgen.1002938.s006.tif]
